# PROSER1 modulates DNA demethylation through dual mechanisms to prevent syndromic developmental malformations

**DOI:** 10.1101/gad.352176.124

**Published:** 2024

**Authors:** Anna Fleming, Elena V. Knatko, Xiang Li, Ansgar Zoch, Zoe Heckhausen, Stephanie Stransky, Alejandro J. Brenes, Simone Sidoli, Petra Hajkova, Dónal O'Carroll, Kasper D. Rasmussen

**Affiliations:** 1 Division of Molecular, Cellular, and Developmental Biology, University of Dundee, Dundee DD1 5EH, United Kingdom;; 2 Centre for Regenerative Medicine, Institute for Regeneration and Repair, Institute for Stem Cell Research, University of Edinburgh, Edinburgh EH16 4UU, United Kingdom;; 3 Wellcome Centre for Cell Biology, University of Edinburgh, Edinburgh EH9 3BF, United Kingdom;; 4 MRC Laboratory of Medical Sciences, London W12 0NN, United Kingdom;; 5 Institute of Clinical Sciences, Faculty of Medicine, Imperial College London, London SW7 2AZ, United Kingdom;; 6 Department of Biochemistry, Albert Einstein College of Medicine, Bronx, New York 10461, USA;; 7 Division of Cell Signalling and Immunology, University of Dundee, Dundee DD1 5EH, United Kingdom

**Keywords:** TET1, TET2, TET3, DNA methylation, TOPD, development, neurodevelopmental disorder

## Abstract

The link between DNA methylation and neurodevelopmental disorders is well established. However, how DNA methylation is fine-tuned—ensuring precise gene expression and developmental fidelity—remains poorly understood. PROSER1, a known TET2 interactor, was recently linked to a severe neurodevelopmental disorder. Here, we demonstrate that PROSER1 interacts with all TET enzymes and stabilizes chromatin-bound TET–OGT–PROSER1–DBHS (TOPD) complexes, which regulate DNA demethylation and developmental gene expression. Surprisingly, we found that PROSER1 also sequesters TET enzymes, preventing widespread demethylation and transposable element derepression. Our findings identify PROSER1 as a key factor that both positively and negatively regulates DNA demethylation essential for mammalian neurodevelopment.

## Abstract (precis)

In this study, Fleming et al. report that PROSER interacts with TET enzymes and stabilizes chromatin-associated TOPD (TET–OGT–PROSER1–DBHS) complexes to maintain genome-wide DNA methylation and repression of endogenous retroviral elements (ERVs). Their findings highlight a prominent role for PROSER in neuronal differentiation and the development of neurodevelopmental disorders.

DNA methylation is a fundamental epigenetic process that is essential for normal development. Collaborating with other chromatin-based epigenetic mechanisms, it safeguards the genome by silencing transposable elements, restricts imprinted gene expression, and balances gene dosage between sexes (Jones 2012). Furthermore, DNA methylation can in some cases directly influence transcription factor binding and gene expression through epigenetic modification of promoter and enhancer regions (Rasmussen et al. 2015, 2019; Schübeler 2015; Kreibich et al. 2023). The TET family of methylcytosine dioxygenases consists of three members (TET1–3). These closely related proteins contain a conserved catalytic domain that can iteratively oxidize 5-methylcytosine (5mC) to 5-hydroxymethylcytosine (5hmC), 5-formylcytosine, and 5-carboxycytosine and promote DNA demethylation (Rasmussen and Helin 2016). Mice lacking all three TET enzymes are unable to survive beyond the early stages of development due to gastrulation failures (Dai et al. 2016), whereas embryos lacking only TET3 can progress to the neonatal stage (Gu et al. 2011). Similarly, although mice lacking either TET1 or TET2 develop normally (Dawlaty et al. 2011; Ko et al. 2011; Li et al. 2011; Moran-Crusio et al. 2011; Quivoron et al. 2011), the combined loss of these enzymes causes developmental abnormalities and increased mortality in a proportion of newborn mice (Dawlaty et al. 2013). The spectrum of developmental defects in knockout mouse lines indicates that TET enzymes have both unique and redundant roles in maintaining developmental processes during early embryonic development.

The function of TET enzymes is modulated via protein–protein interactions with a diverse set of binding partners. For instance, TET enzymes interact with O-linked N-acetylglucosamine (O-GlcNAc) transferase (OGT) (Chen et al. 2013; Deplus et al. 2013; Vella et al. 2013) and the SIN3A–HDAC complex (Williams et al. 2011; Zhang et al. 2015; Zhu et al. 2018; Flores et al. 2023) to promote histone O-GlcNAcylation and histone deacetylation, respectively. In addition, TET1 and TET2 interact with the two *Drosophila* behavior/human splicing (DBHS) proteins Paraspeckle component 1 (PSPC1) and non-POU domain-containing octamer binding (NONO) (Knott et al. 2016, 2022) to modulate expression of endogenous retroviruses and bivalent genes (Guallar et al. 2018; Li et al. 2020; Huang et al. 2022). Finally, TET2 has recently been reported to interact with proline- and serine-rich 1 (PROSER1) in the context of UTX and the MLL3/4 branch of COMPASS (complex of proteins associated with SET1) to modulate H3K4me1 and H3K4me2 levels at UTX binding sites in the human embryonic kidney cell line HEK293 (Wang et al. 2022). The relative importance of these interactions in regulating TET activity and function is an active area of research.

Interestingly, recent findings have linked homozygous loss-of-function mutations in PROSER1 to a novel developmental disorder. This condition features hypotonia, developmental delays, genitourinary malformations, and craniofacial abnormalities associated with sensorineural hearing loss and strabismus (Salah et al. 2022). Given the established role of DNA methylation in neurodevelopment, as evidenced by the association of mutations in DNMT1, DNMT3A, DNMT3B, USP7, and TET3 with a wide spectrum of developmental disorders (Nava and Arboleda 2024), the observed interaction between PROSER1 and TET2 is intriguing. Although this interaction suggests a potential mechanism underlying PROSER1-associated syndromes, the precise pathological consequences of PROSER1 deficiency on DNA methylation, gene expression, and ultimately developmental integrity during early embryogenesis remain to be elucidated.

## Results and Discussion

### Loss of PROSER1 increases preweaning lethality and is associated with developmental disabilities and craniofacial abnormalities

**Figure 1.**
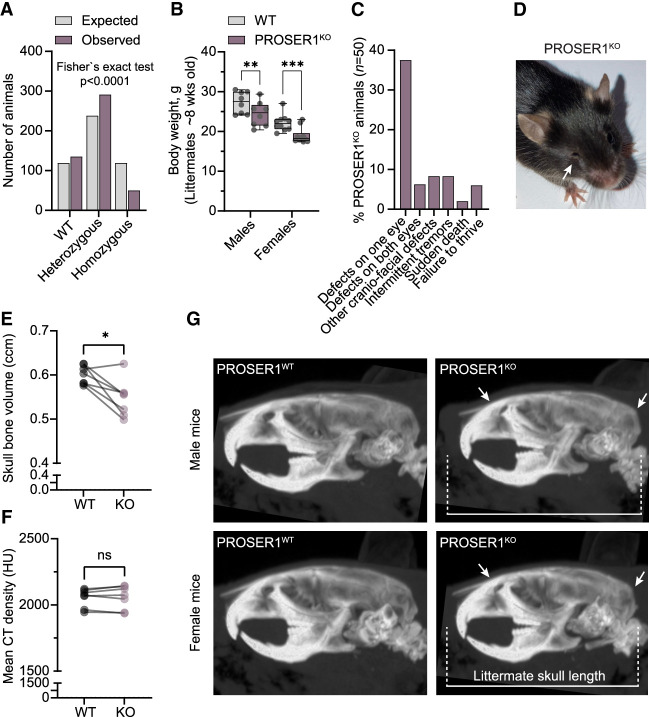
Loss of PROSER1 increases preweaning lethality and is associated with developmental disabilities and craniofacial abnormalities. (*A*) Histogram showing distribution of genotypes at weaning for 476 pups born from heterozygous *Proser1*^+/−^ breeder pairs. The number of homozygous PROSER1 knockout pups at weaning is demonstrably lower than expected, with <50% being recovered. Statistical significance for the contingency table was measured by Fisher's exact test. *P* < 0.0001. (*B*) Box plot showing the body weight of adult PROSER1 knockout (KO) and wild-type (WT) littermates. Comparisons were made for males and females separately. *n* = 8–10. Statistical significance was measured using two-way ANOVA with Sidàk multiple comparisons correction. (**) *P* < 0.01, (***) *P* < 0.001. (*C*) Bar chart showing percentages of gross abnormalities observed in adolescent and adult PROSER1 knockout animals. *n* = 50. Eye defects include microphthalmia, anophthalmia, corneal ulcers, and cataracts. Other craniofacial defects include intermittent head tilt and distorted ear (indicative of otitis media), as well as one case of suspected hydrocephalus. (*D*) Image of a representative PROSER1 KO animal with microphthalmia. The affected eye is indicated by an arrow. (*E*) Dot and line graph representing volumetric analysis of microcomputed tomography (microCT) scans of PROSER1 KO and wild-type littermate skulls. *n* = 7. Statistical significance was measured by paired two-tailed *t*-test (*) *P* < 0.05. (*F*) Same as *E* but representing average CT density (Hounsfield units) as an indication of bone mineral density. (ns) Not significant. (*G*) Lateral views of representative microCT scans of a 13 week old male (*top*) and 14 week old female (*bottom*) PROSER1 KO animal as well as wild-type littermates. The images are rendered as maximum intensity projections (MIPs) from an equal-sized volume to enable direct visual comparison between specimens. Arrows indicate malformations of maxilla and frontal bones as well as rounded head shape. Scale bar indicates skull length of wild-type littermates.

To establish a direct causal link between PROSER1 loss-of-function gene mutations and neurodevelopmental disorders, we generated a mouse line with constitutive inactivation of the endogenous *Proser1* gene using CRISPR–Cas9 gene editing (Supplemental Fig. S1A–C). *Proser1*^+/−^ mice were viable and fertile, and upon backcrossing to C57BL/6J, these mice were intercrossed to generate mice with homozygous PROSER1 loss. Analysis of offspring from these breeders demonstrated that PROSER1 knockout results in partially penetrant preweaning lethality (Fig. 1A). We did not observe prominent increases in perinatal lethality, suggesting that most PROSER1 knockout embryos may be reabsorbed in utero during early gestation. Surviving PROSER1 knockout animals weighed less upon reaching adulthood (Fig. 1B) and displayed frequent eye abnormalities, including microphthalmia, anophthalmia, and cataracts, as well as intermittent tremors and failure to thrive (Fig. 1C,D). To further characterize neuroanatomical defects, we performed microcomputed tomography (microCT) scanning of skulls from adult animals (8–14 weeks old). Consistent with reduced overall weight, volumetric analysis revealed reduced cranial bone volume in PROSER1 knockout animals compared with wild-type littermates (Fig. 1E), while bone density remained unchanged (Fig. 1F). Of note, the persistence of this phenotype in fully grown animals suggests that loss of PROSER1 results in permanent developmental disability rather than developmental delays. Further comparison of skull shape revealed distinct malformations of the maxillary and frontal bones as well as a tendency for rounded heads and a general shortening of the skull (Fig. 1G). In summary, germline PROSER1 deficiency in mice results in pleiotropic developmental abnormalities that resemble the human neurodevelopmental disorder in which PROSER1 is mutated.

### PROSER1 is a pan-TET interactor and participates in chromatin-associated TOPD complexes

**Figure 2.**
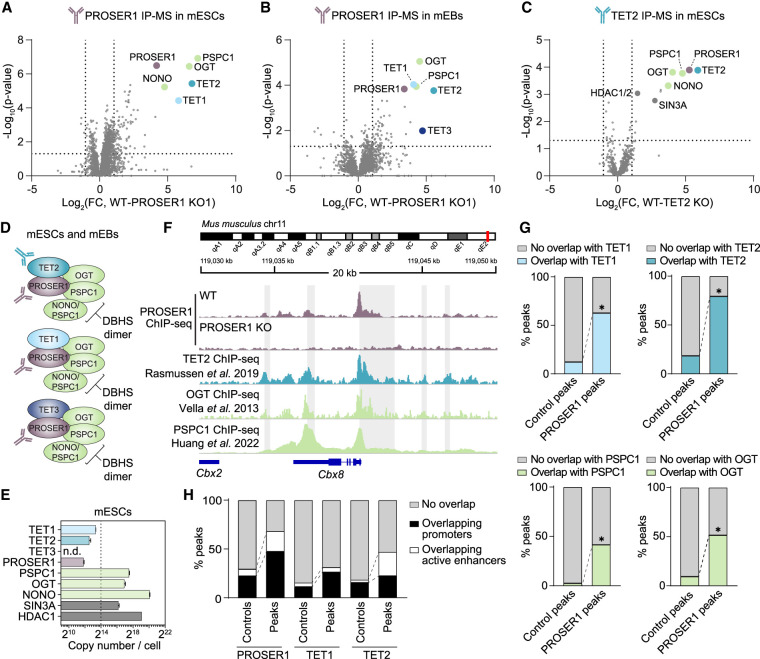
PROSER1 is a pan-TET interactor and participates in chromatin-associated TOPD complexes. (*A*) Volcano plot showing protein hits from α-PROSER1-C immunoprecipitation and mass spectrometry (IP-MS) in WT and PROSER1 KO mESCs. *n* = 3 biological replicates. Dotted lines indicate twofold change and *P*-value of 0.05. Proteins of interest are highlighted and identified. (*B*) As in *A* but carried out in cells differentiated for 2 days to mouse embryoid bodies (mEBs) where TET3 expression is high compared with mESCs. (*C*) As in *A* but carried out using α-TET2-N antibody and protein enrichment compared with parallel IP-MS in WT and TET2 KO mESCs. See Supplemental Tables S3–S6 for full lists of enriched proteins in the IP-MS experiments. (*D*) Illustration of the potential composition of TOPD complexes in mESCs and mEBs. The DBHS protein dimer is depicted as consisting of PSPC1 and NONO for simplicity but may be variable in vivo. (*E*) Copy number estimation (Wiśniewski et al. 2014) of TET proteins and TET interactors in mESCs determined by MS. *n* = 3 biological replicates. The dotted line indicates the sum of TET1, TET2, and TET3 copies. Error bars represent the mean ± SD. (*F*) ChIP-seq tracks showing a representative region (around the *Cbx8* gene) bound by PROSER1, TET2, OGT, and PSPC1 in WT mESCs. CGIs are indicated in gray. (*G*) Percentage of PROSER1-N peaks or matched controls (matched to the PROSER1-N peak set in number, size, and distance to DNase hypersensitivity sites in mESCs and generated using Easeq) (Lerdrup et al. 2016) that overlap with TET1, TET2, PSPC1, or OGT binding. (*) *P* < 0.0001, two-tailed Fisher's exact test. (*H*) Percentage of PROSER1-N, TET1, or TET2 peaks or matched controls that overlap with active enhancers or promoters. All peak sets/tracks except PROSER1 were generated from publicly available data (Williams et al. 2011; Vella et al. 2013; Rasmussen et al. 2019; Huang et al. 2022).

Our data demonstrate functional homology between mouse and human PROSER1, suggesting that mouse embryonic stem cells (mESCs) and their differentiation can serve as an appropriate and tractable model system to elucidate the mechanistic role of PROSER1 in early development. A previous study reported a protein–protein interaction between PROSER1 and TET2 in HEK293 cells (Wang et al. 2022). To determine the conservation of this interaction in mESCs and its potential extension to the entire TET family of enzymes, we raised two specific antimurine PROSER1 antibodies with epitopes from PROSER1 C and N termini, respectively, and performed endogenous IP-MS using PROSER1 knockout mESCs as background controls (Fig. 2A; Supplemental Fig. S1D–F; Supplemental Tables S1, S2). Analysis of biological triplicate experiments identified significant enrichment of TET1 and TET2 as well as the previously identified TET protein interactors OGT, PSPC1, and NONO. These interactions were also observed in protein lysates isolated from mESC-derived mouse embryoid bodies (mEBs). Unlike mESCs, mEBs express TET3, and indeed we observed robust enrichment of all three TET enzymes upon PROSER1 IP (Fig. 2B). Importantly, by performing TET2 IP-MS in wild-type and TET2 knockout mESCs, we could further recover PROSER1, OGT, PSPC1, and NONO interactions but no detectable interaction with the other TET enzymes (Fig. 2C). These findings indicate that the presence of TET2 in PROSER1-containing complexes is mutually exclusive with TET1 and TET3, suggesting that PROSER1 forms discrete complexes with each TET protein. Indeed, PROSER1 IP-MS in TET2 knockout cells still robustly enriched TET1 and TET3, demonstrating that their interaction with PROSER1 is not dependent on TET2 (Supplemental Fig. S2A).

Our data suggest the existence of multiprotein complexes involving TET proteins, OGT, and PROSER1, as well as members of the DBHS family, which are referred to here as TOPD (TET–OGT–PROSER1–DBHS proteins) complexes (Fig. 2D). The relative abundance of these complexes is likely affected by variation in the expression of TET proteins and their interactors in different tissues. As mentioned above, TET1 and TET2 are highly expressed in mESCs, whereas TET3 expression is induced in mEBs and upon differentiation to neuronal lineages. Similarly, although the DBHS protein PSPC1 robustly associates with TET complexes in all cells tested, PSPC1 can form either PSPC1–PSPC1 homodimers or PSPC1–NONO and PSPC1–SFPQ heterodimers, depending on the relative abundance of each protein (Knott et al. 2016, 2022). Of note, estimation of absolute protein abundance in wild-type mESCs shows that TET proteins and PROSER1 are present at similar copy numbers, whereas the abundance of OGT, PSPC1, and NONO is orders of magnitude higher (Fig. 2E). This implies that excess OGT, PSPC1, and NONO is likely to be involved in processes independent of TET proteins and PROSER1. In contrast, most of the cellular pool of PROSER1 may be engaged within TOPD complexes. Interestingly, PROSER1 IP-MS did not result in enrichment of SIN3A or HDAC1/2 (Fig. 1A,B; Supplemental Table S3), suggesting that TET interactions with the SIN3A–HDAC complex are independent of PROSER1. We also failed to detect interactions with UTX or members of COMPASS, and profiling of histone modifications by quantitative mass spectrometry revealed little or no global changes in H3K4 methylation in two independent PROSER1 knockout mESC lines (Supplemental Fig. S1G).

Initial biochemical cell fractionation of mESCs demonstrated that PROSER1 is predominantly a chromatin-associated protein (Supplemental Fig. S2B). We therefore performed PROSER1 chromatin immunoprecipitation and sequencing (ChIP-seq) using our antimurine PROSER1-N antibody to gain further insights into its function in chromatin. To ensure specificity of enriched peaks, ChIP-seq was carried out simultaneously on wild-type and PROSER1 KO mESCs. Analysis of biological replicate experiments revealed 1712 high-confidence PROSER1 binding sites (Supplemental Fig. S2C). Upon intersection with publicly available ChIP-seq data sets in mESCs, we found that a large majority (>95%) are co-occupied by TET1, TET2, OGT, PSPC1, or combinations of these (Fig. 2F; Supplemental Fig. S2D). We next asked whether PROSER1 genome colocalization with each of the complex components is enriched compared with matched control regions. We observed significant enrichment (*P* < 0.0001, Fisher's exact test) for TET1, TET2, OGT, and PSPC1 colocalization (Fig. 2G), whereas no enrichment was observed at CTCF binding sites or gene bodies (Supplemental Fig. S2E,F). Notably, genomic regions associated with binding of both TET1 and TET2 were significantly enriched within PROSER1 peaks (51.7% vs. 10.5% in the rest of the genome; *P*-value < 0.0001, Fisher's exact test) (Supplemental Fig. S2G), suggestive of their interchangeable roles within TOPD complexes. Consistent with a role in gene regulation, PROSER1 high-confidence binding sites are associated with both active (H3K27ac and P300) and repressive (H3K27me3 and SUZ12) chromatin domains (Supplemental Fig. S2H) and overlap regulatory genomic regions such as promoters, CpG islands (CGIs), and active enhancers that are known to be occupied by TET1 and TET2 (Fig. 2H; Williams et al. 2011; Rasmussen et al. 2019).

### PROSER1 loss disrupts TOPD complexes and alters TET2 genome-wide chromatin binding

**Figure 3.**
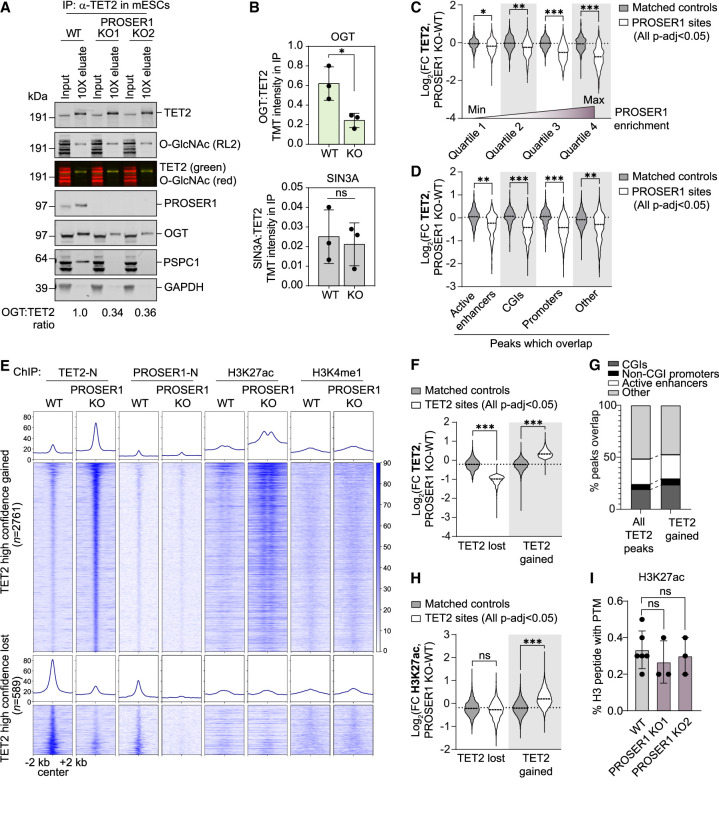
PROSER1 loss disrupts TOPD complexes and alters TET2 genome-wide chromatin binding. (*A*) Western blot of input and 10× concentration eluates from α-TET2 IP on lysates from WT, PROSER1 KO1, and PROSER1 KO2 mESCs. The intensity of OGT in each eluate was normalized to the intensity of TET2 in the same eluate, and the ratios in each IP are provided *below* the figure. A slight difference in apparent molecular weight was observed between inputs and eluates owing to different buffer compositions. (*B*) Ratios of OGT:TET2 and SIN3A:TET2 in TMT-labeled quantitative mass spectrometry analysis of TET2 immunoprecipitate in WT and PROSER1 KO mESCs. TMT reporter intensity was quantified from three biological replicates. Error bars represent the mean ± SD. (*) *P* < 0.05 (unpaired two-tailed *t*-test with Welch's correction), (ns) nonsignificant. (*C*) Fold change in TET2 binding based on TET2 normalized read counts within PROSER1 peaks or matched control regions upon PROSER1 loss. PROSER1 peaks were sorted by log_2_ (fold change in PROSER1 and KO WT) and divided into equal-sized quartiles (white). Controls (gray) were generated for each quartile. The effect sizes of binding loss compared with matched control regions were measured with Cohen's *d*. (*) *d* > 0.3 (small effect), (**) *d* > 0.6 (medium effect), (***) *d* > 0.9 (large effect). (*D*) As in *C*, but TET2 normalized read counts are shown at PROSER1 sites within different genomic regions (white), as specified *below* the plot, or controls (gray) generated for the set of PROSER1 peaks within each genomic region. (*E*) Heat maps and mean values of normalized ChIP-seq signals for TET2, PROSER1, H3K27ac, and H3K4me1 centered at high-confidence [*P*-adj < 0.05, abs(fold change ≥ 2)] sites with gain or loss of TET2 binding. Regions are ranked on *P*-adj values for TET2 binding between PROSER1 KO and WT. Heat maps were generated using DeepTools software (Ramírez et al. 2014). (*F*) As in *C* but showing fold change in TET2 binding based on TET2 normalized read counts within all significant (*P*-adj < 0.05) TET2 differential sites upon PROSER1 loss. (*G*) Percentage of all TET2 peaks (Rasmussen et al. 2019) or all sites with significant (*P*-adj < 0.05) gain of TET2 binding that overlap with genomic regions as specified *above* the plot. (*H*) As in *F* but showing fold change in H3K27ac. (*I*) Global enrichment of H3K27ac in WT, PROSER1 KO1, and PROSER1 KO2 mESCs as measured by quantitative MS. *n* = 3 biological replicates of PROSER1 KO lines, *n* = 6 biological replicates of WT. Error bars represent the mean ± SD. Statistical significance was measured by unpaired two-tailed *t*-test with Welch's correction.

To understand how loss of PROSER1 affects the stability of TOPD complexes, we immunoprecipitated TET2 in wild-type and PROSER1 knockout mESC lines and analyzed eluates by Western blotting and label-free mass spectrometry (Fig. 3A; Supplemental Fig. S3A). Although PROSER1, OGT, and PSPC1 were present in TET2 eluates from wild-type cells, loss of PROSER1 reduced the relative recovery of OGT and abolished TET2–PSPC1 interactions altogether. Importantly, proteomic analysis of the knockout cell lines demonstrated that the expression levels of TET2, OGT, and PSPC1 were unaffected, indicating that the loss of interaction is not a consequence of reduced protein stability (Supplemental Fig. S3B). Follow-up analysis of biological triplicate IP eluates using quantitative TMT labeling demonstrated that TET2–OGT interactions were reduced by ∼60% upon loss of PROSER1, while quantitative recovery of SIN3A was not affected (Fig. 3B). Of note, we found that the level of TET2 O-GlcNAcylation was unchanged in PROSER1 knockout cells, suggesting that enzyme–substrate interactions between TET2 and OGT are preserved in the absence of PROSER1 (Fig. 3A).

To investigate the association between PROSER1, TOPD recruitment to chromatin, and the activation status of PROSER1-bound regions, we then performed ChIP-seq for TET2 as well as the histone marks H3K4me1 and H3K27ac in wild-type and PROSER1 knockout mESCs (Supplemental Fig. S3C). Differential enrichment analysis within high-confidence PROSER1 binding sites revealed that loss of PROSER1 correlates with reduced TET2 chromatin occupancy, whereas enrichment of H3K4me1 or H3K27ac does not change in response to loss of PROSER1 within the same regions (Fig. 3C; Supplemental Fig. S3D–F). We observed that TET2 binding loss is not limited to PROSER1 binding sites within specific regulatory domains but rather is seen across active enhancers, promoters, and CGIs (Fig. 3D). In contrast to the reduction of TET2 binding at PROSER1-bound genomic regions, we also observed a significant number of sites with increased TET2 occupancy upon knockout of PROSER1 (Fig. 3E,F). These regions do not show evidence of PROSER1 binding in wild-type cells (Fig. 3E; Supplemental Fig. S3F) or skewing toward increased association with specific genomic regions compared with TET2 binding sites in wild-type cells (Fig. 3G). However, these regions were linked to increases in H3K27ac upon PROSER1 knockout (Fig. 3E,H; Supplemental Fig. S3G), possibly via interactions of TET2 with the histone acetyltransferase P300, as reported previously (Zhang et al. 2017). Of note, we did not observe global differences in P300-directed histone acetylation via histone mass spectrometry or local differences at matched control regions that were not bound by TET2, suggesting that increased H3K27ac deposition is largely restricted to sites associated with increased TET2 binding (Fig. 3H,I; Supplemental Fig. S3H). Collectively, our results demonstrate that PROSER1 is required for the stability of TOPD protein complexes, and that loss of PROSER1 alters the recruitment of TET2 to chromatin.

### PROSER1 knockout unleashes TET catalytic activity and causes widespread DNA demethylation and desilencing of endogenous retroviruses

**Figure 4.**
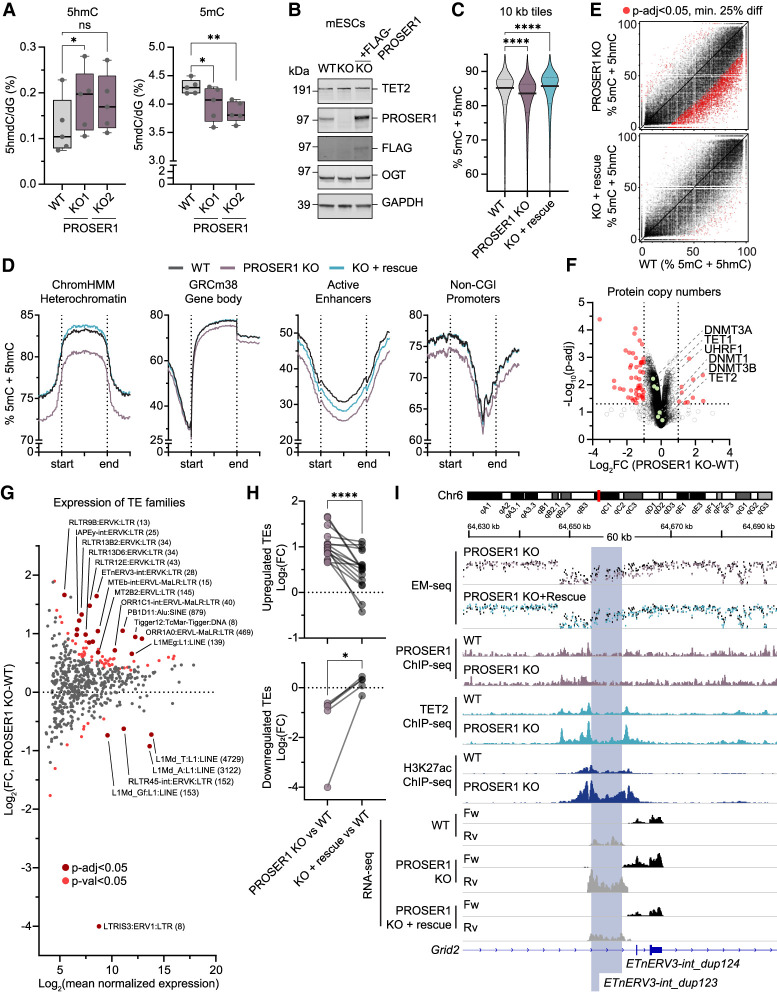
PROSER1 loss unleashes TET activity and causes widespread DNA demethylation and derepression of endogenous retroviruses. (*A*) Quantification of 5hmC and 5mC by LC-MS/MS in genomic DNA harvested from WT, PROSER1 KO1, and PROSER1 KO2 mESCs. Each symbol represents a sample harvested and processed independently (*n* = 5 biological replicates with each two technical replicates) from each cell line. Statistical significance was measured by paired two-tailed *t*-test (paired by same day of harvest due to coordinated fluctuations in 5hmC). (ns) Not significant, (*) *P* < 0.05, (**) *P* < 0.01. (*B*) Western blot of lysates prepared from WT, PROSER1 KO, and PROSER1 KO + rescue mESCs. GAPDH was probed as a loading control. (*C*) Violin plot showing average DNA methylation in 10 kb tiles for CpG sites covered by a minimum of 10 EM-seq reads in all samples in WT, PROSER1 KO, and PROSER1 + rescue mESCs. Lines on violin plot represent the median and quartiles. Statistical significance was measured by Brown–Forsythe and Welch ANOVA test. (****) *P* < 0.0001. (*D*) Quantitation trend plots of DNA methylation quantified by EM-seq in heterochromatin, gene bodies, active enhancers, and non-CGI promoters for CpG sites covered by a minimum of 10 EM-seq reads in all samples in WT, PROSER1 KO, and PROSER1 KO + rescue mESCs. (*E*) XY scatter plot showing the modification state of individual CpG sites covered by at least 10 EM-seq reads that overlap 3196 regions with increased H3K27ac deposition identified previously (Supplemental Fig. S3B) in PROSER1 KO versus WT cells. Significantly differentially methylated CpG sites (*P*-adj < 0.05; minimum 25% difference) are highlighted in red for PROSER1 KO versus WT (*top*) and PROSER1 + rescue versus WT (*bottom*). (*F*) Volcano plot showing protein copy numbers determined by whole-proteome MS on wild-type and PROSER1 KO mESCs. *n* = 3 biological replicates. Dotted lines indicate twofold change and *P*-adj 0.05. Components of the DNA methylation machinery are highlighted and identified. (*G*) MA-plot showing TEtranscripts differential expression analysis of TEs in WT and PROSER1 KO mESCs. Each dot represents a subfamily of TE elements, and the number of individual elements included in the analysis (covered by at least one unique read) is shown in parentheses. Red and dark-red dots indicate significantly differentially expressed TE families at thresholds of *P*-value < 0.05 and *P*-adj < 0.05, respectively. (*H*) Symbol and line plots comparing expression of TE families in PROSER1 KO versus WT and PROSER1 KO + rescue versus WT. Plots include TE families found to be differentially expressed (*P*-adj < 0.05) between WT and PROSER1 KO in *G* and split to show upregulated (*top*) and downregulated (*bottom*) TE families. Statistical significance was measured by paired two-tailed *t*-test (*) *P* < 0.05, (****) *P* < 0.0001. (*I*) Tracks showing regions surrounding *ETnERV3-int_dup123* and *ETnERV3-int_dup124* (highlighted in blue) that gain TET2 binding and H3K27ac and become DNA hypomethylated and desilenced in the absence of PROSER1. Data in the *top* two panels represent pooled EM-seq methylation calls. Data from WT mESCs are shown in black, and data from PROSER1 KO and PROSER1 KO + rescue are overlaid in purple or blue, respectively. (*Bottom*) Tracks represent ChIP-seq coverage in PROSER1 ChIP-seq (*n* = 2 biological replicates) and TET2 or H3K27ac ChIP-seq (*n* = 3 biological replicates. The last six tracks represent coverage of forward (Fw) or reverse (Rv) transcripts identified by RNA-seq. *n* = 2 biological replicates.

To determine the effect of PROSER1 loss on DNA methylation, we harvested genomic DNA from two independent PROSER1 knockout mESC lines and quantified global levels of 5hmC and 5mC by mass spectrometry. We observed a decrease in global 5mC levels as well as slightly elevated levels of genomic 5hmC, the major product of TET catalytic activity (Fig. 4A). To investigate which regions are affected by increased TET activity, we generated base-resolution DNA methylation profiles using enzymatic methyl-sequencing (EM-seq) in wild-type ESCs (WT), PROSER1 knockout (PROSER1 KO) cells, and PROSER1 knockout cells that were engineered to re-express full-length FLAG-tagged PROSER1 (PROSER1 KO + rescue) (Fig. 4B). Mapping of EM-seq reads to the mouse genome allowed quantification of cytosine modification states at ∼17.5 million CpG sites with at least 10× coverage in all three genotypes. Consistent with our mass spectrometry results, we observed a decrease in DNA methylation in 10 kb windows across the entire genome in PROSER1 KO cells, whereas reintroduction of PROSER1 restored methylation to wild-type levels (Fig. 4C). This widespread DNA hypomethylation was also observed when comparing average DNA methylation levels in diverse genomic regions including heterochromatin, gene bodies, active enhancers, and non-CGI promoters (Fig. 4D), though it was noted that regions generally depleted of DNA methylation, such as CGIs and bivalent promoters, were unchanged (Supplemental Fig. S4A). We furthermore found significant DNA hypomethylation at sites associated with increased H3K27ac deposition identified previously (Fig. 4E; Supplemental Fig. S3C). Importantly, expression and protein copy numbers of the major DNA methylation effectors (DNMT1/UHRF1, DNMT3A/B, TET1, and TET2) were largely unchanged (Fig. 4F; Supplemental Fig. S4B). This implies that DNA methylation changes are a direct consequence of altered TET activity in PROSER1 knockout cells rather than a result of a general disruption of DNA methylation maintenance machinery.

Dual pharmacological inhibition of DNA methylation enzymes and histone deacetylases causes DNA hypomethylation and increased histone acetylation—reminiscent of changes observed upon PROSER1 knockout—and synergizes to cause desilencing of transposable elements (TEs) (Brocks et al. 2017; Daskalakis et al. 2018; Cusack et al. 2020; Goyal et al. 2023). We therefore used a combination of RNA-seq and TEtranscripts, an analysis pipeline designed to handle reads that map to multiple locations in the genome, to assign multimapping reads to specific TE families and analyze their activity. Loss of PROSER1 led to an increase in transcription of multiple families of long terminal repeat (LTR)-containing endogenous retroviral (ERV) elements such as ERVK, ERVL, and ERVL-MaLR, whereas expression of the non-LTR L1Md retrotransposons was mildly reduced (Fig. 4G). To understand whether this deregulation correlated with loss of PROSER1, we assessed transcript levels of the differentially expressed TE families upon re-expression of PROSER1. Consistent with the observed restoration of DNA methylation levels described above, expression of differentially expressed TE families (Fig. 4H) as well as expression of individual TE elements identified solely based on uniquely mapped reads (Fig. 4I; Supplemental Fig. S4C) were restored to near wild-type levels upon reintroduction of full-length PROSER1. Collectively, our findings demonstrate that PROSER1 safeguards against genome-wide DNA demethylation and aberrant activation of endogenous retroviruses.

### Defective recruitment of TET2 to developmental genes upon PROSER1 loss leads to their subsequent dysregulation during neuronal differentiation

**Figure 5.**
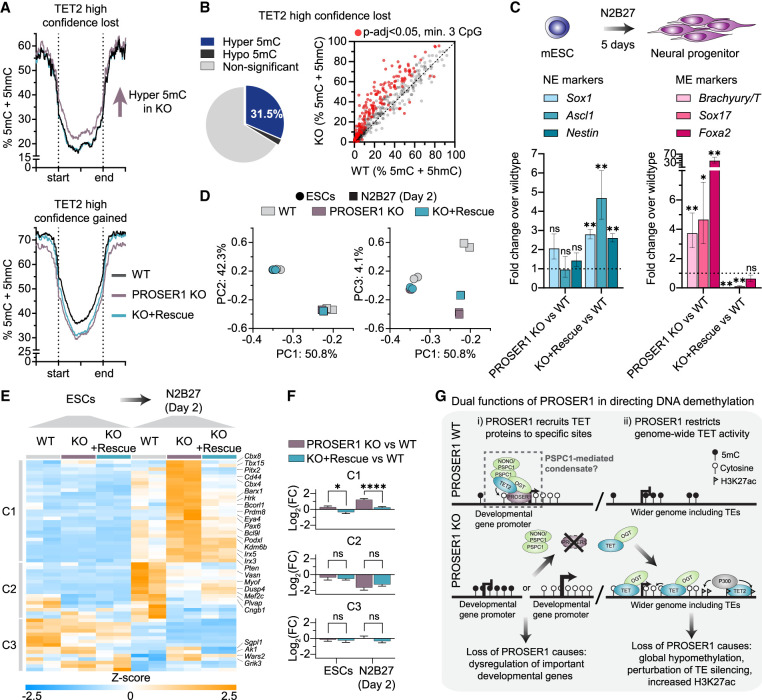
Defective recruitment of TET2 to developmental genes upon PROSER1 loss leads to their subsequent dysregulation during neuronal differentiation. (*A*) Quantitation trend plots of DNA methylation for CpG sites covered by a minimum of 10 EM-seq reads in WT, PROSER1 KO, and PROSER1 KO + rescue mESCs at high-confidence [*P*-adj < 0.05, abs(fold change ≥ 2)] sites with loss (*top*) or gain (*bottom*) of TET2 binding upon PROSER1 KO. (*B*) Pie chart (*left*) and XY scatter plot (*right*) showing average DNA methylation within high-confidence sites with loss of TET2 binding upon PROSER1 KO. Significantly (*P*-adj < 0.05) hypermethylated or hypomethylated sites (minimum of three CpGs per site; minimum of 10 reads per CpG) are indicated. (*C*) Expression of neuroectodermal (NE) or mesendodermal (ME) markers upon 5 days of differentiation of WT, PROSER1 KO, and PROSER1 KO + rescue mESCs to neuronal progenitors. Bars represent fold change of expression compared with wild-type neural progenitors. Error bars represent the estimated standard error (StdErr) from DESeq2 output. Statistical significance was measured by Benjamini–Hochberg adjusted *P*-value. (*) *P*-adj < 0.05, (**) *P*-adj < 0.001, (ns) nonsignificant. (*D*) PCA plot of RNA-seq data. PCA was carried out using *Z*-scores of the top 2500 variable genes in WT, PROSER1 KO1, and PROSER1 KO + rescue cells in the mESC state (circles) or after 2 days of differentiation toward neuronal progenitors (squares). (*E*) Heat map showing *Z*-scores of genes differentially expressed at day 2 of neuronal differentiation between PROSER1 KO and WT (*P*-adj < 0.05) with regulatory domains overlapping high-confidence sites with loss of TET2 binding upon PROSER1 KO in mESCs. Hierarchical clustering identified three distinct clusters (C1–C3) as indicated. See Supplemental Table S7 for a full list of differentially expressed genes associated with loss of TET2 binding. (*F*) Bar charts showing average log_2_ fold change for each individual cluster defined in *E* for PROSER1 KO versus WT and PROSER1 + rescue versus WT in mESCs and after 2 days of neuronal differentiation, respectively. Statistical significance was measured by Brown–Forsythe and Welch ANOVA test. (*) *P* < 0.05, (****) *P* < 0.0001. (*G*) Schematic illustrating the dual roles of PROSER1 in directing TET function and DNA demethylation.

To determine the direct effect of altered TET2 chromatin binding, we quantified DNA methylation changes in the high-confidence TET2 differentially bound sites [*P*-adj < 0.05, abs(fold change) ≥ 2] identified in PROSER1 knockout cells (Fig. 3E). In contrast to regions with gained TET2 binding, which mirrored the genome-wide DNA hypomethylation, sites with reduced TET2 binding were instead correlated with increased levels of DNA methylation (Fig. 5A). Further analysis identified that nearly one-third of sites with reduced TET2 binding exhibited a significant rise in average DNA methylation (*P*-adj < 0.05; minimum of three CpGs per site) (Fig. 5B). To investigate potential effects on gene expression, we identified enriched gene ontology terms in the subset of 866 genes whose regulatory domains (defined in GREAT as “basal plus extension”) (McLean et al. 2010) overlapped with sites with reduced TET2 binding. This analysis revealed significant associations with early development processes, such as nervous and skeletal system development, and was linked to mouse knockout phenotypes exhibiting craniofacial abnormalities including eye defects (Supplemental Fig. S4D). In contrast, genes whose regulatory domains overlapped with sites with increased TET2 binding were largely linked to phenotypes associated with abnormal hematopoietic differentiation (Supplemental Fig. S4E).

To directly assess the effect of PROSER1 knockout during mESC differentiation, we differentiated mESCs toward neuronal progenitors using the N2B27 monolayer differentiation system (Ying et al. 2003). By day 5 of differentiation, a time point by which most cells have acquired a neuronal cell fate and key neuronal markers are significantly upregulated, gene expression analysis of PROSER1 knockout and wild-type cultures revealed comparable levels of neuroectodermal (NE) markers (e.g., *Sox1*, *Ascl1*, and *Nestin*), whereas mesendodermal (ME) markers (e.g., *Brachyury/T*, *Sox17*, and *Foxa2*) were upregulated in PROSER1 knockout cells. Conversely, ectopic expression of FLAG-tagged PROSER1 during differentiation (PROSER1 KO + rescue) reversed this phenotype, characterized by an increase of NE markers and a concomitant decrease of ME markers (Fig. 5C). This suggests that PROSER1 may play a role in suppressing mesendodermal development and facilitate faithful execution of the NE gene expression program during early lineage specification. To gain further insights, we analyzed gene expression changes at day 2 following induction of differentiation, a time point chosen to ensure strong correlation between ChIP occupancy data generated in mESCs and gene expression changes. At this early stage of differentiation, principal component analysis (PCA) revealed differentiation state as the main factor driving variation in the samples (PC1: 50.8%; PC2: 42.3%). In addition, we identified a minor component (PC3: 4.1%) that correlated with PROSER1 expression and separated samples cultured for 2 days in N2B27 medium (Fig. 5D). We therefore examined whether the reduced TET2 chromatin binding that we observed in PROSER1 knockout mESCs could be linked to gene expression changes at this developmental stage. To do this, we focused on a subset of 60 genes that (1) had a regulatory domain overlapping sites of reduced TET2 binding in mESCs, and (2) were differentially expressed (*P*-adj < 0.05) between wild-type and PROSER1 knockout cells after 2 days of neuronal differentiation (Fig. 5E; Supplemental Table S7). Contingency table analysis revealed significant enrichment of this subset of genes (both increased and decreased in knockout) compared with the subset of differentially expressed genes not associated with reduced TET2 binding sites (7.43% vs. 3.91% of total; *P*-value < 0.0001, Fisher's exact test). Hierarchical clustering identified three clusters (C1–C3) with different expression patterns broadly classified as upregulation, downregulation, or mixed regulation in PROSER1 knockout cells, respectively (Fig. 5E). The observed increases and decreases in transcript levels upon PROSER1 depletion suggest that TOPD complexes possess both activating and repressive regulatory capacities, the specific outcome of which is likely influenced by genomic location and developmental stage. Importantly, comparison of log_2_ fold changes in PROSER1 KO versus WT and in PROSER1 KO + rescue versus WT showed that reintroduction of PROSER1 restored expression of many of these genes, particularly in C1, to near wild-type levels (Fig. 5F). Genes cobound by PROSER1/TET2 and whose expression correlated with PROSER1 expression included important developmental regulators such as homeobox genes (e.g., *Pitx2*, *Barx1*, and *Pax6*), epigenetic regulators (e.g., *Cbx4*, *Cbx8*, and *Prdm8*), and transcription factors (e.g., *Tbx15* and *Mef2c*), implying that PROSER1 plays a regulatory role in their function during early embryonic development (Supplemental Fig. S5A–C). We further noted significant deregulation of Eyes absent homolog 4 (*Eya4*), a PROSER1/TET2 cobound target gene that is crucial for eye, heart, and sensorineural development (Supplemental Fig. S5A–C; Tadjuidje and Hegde 2013). Collectively, deregulation of these genes may underlie some, if not all, of the neurodevelopmental defects observed upon loss of PROSER1 in mice and humans.

In summary, our results are consistent with a dual role for PROSER1 in directing DNA demethylation in early development (Fig. 5G). We show that PROSER1 is a pan-TET interactor that promotes the assembly of TOPD protein complexes and facilitates their recruitment to chromatin in the proximity of important developmental genes. The recruitment of TET proteins, particularly TET2, at PROSER1-bound sites maintains a lowly methylated state at regulatory regions and appropriate expression of adjacent genes during differentiation. Our findings also indicate that TOPD complexes sequester TET2 away from other regions of the genome. When PROSER1 is depleted, TET2 binds to additional sites, resulting in widespread DNA demethylation and therefore global DNA hypomethylation. Interestingly, the regions with increased TET2 binding also displayed increased levels of H3K27 acetylation. This suggests that P300, a histone acetyltransferase previously shown to interact with TET2 (Zhang et al. 2017), might be recruited alongside TET2 to these same sites. The combined effects of reduced DNA methylation and increased chromatin openness (caused by P300 activity) cooperate to perturb the silencing of endogenous retroviral elements, potentially disrupting the expression of adjacent genes during differentiation. Although our analysis has focused on TET2, the overlapping binding pattern of TET1 and TET2 within PROSER1 binding sites (Fig. 2G; Supplemental Fig. S2G) and the shared features of TET proteins and TOPD complexes (Fig. 2A–C; Supplemental Fig. S2A) make it plausible that all three TET enzymes exhibit similar binding dynamics upon PROSER1 knockout in pluripotent and differentiating mESCs (Fig. 5G). However, their specific roles in the observed phenotypes require further investigation.

The exact mechanism by which TOPD complexes control TET activity across the genome remains unclear. One hypothesis is that certain PROSER1 binding sites act as “sponges,” attracting TOPD complexes and TET proteins to specific genomic sites and preventing widespread, uncontrolled DNA demethylation by TET enzymes. Another interesting possibility involves the RNA-binding TOPD component PSPC1, which we have found to interact with TET2 in a PROSER1-dependent manner (Fig. 3A; Supplemental Fig. S3A). DBHS proteins including PSPC1 can form higher-order oligomers (Knott et al. 2016, 2022; Fox et al. 2018), potentially functioning as RNA- or DNA-tethered condensates that sequester TOPD complexes, further regulating TET activity. Of note, cell fractionation demonstrated that TET2 subcellular localization remains largely unaltered in PROSER1 knockout mESCs (Supplemental Fig. S2B), suggesting that changes associated with PROSER1 loss occur without a significant redistribution of TET2 between biochemically defined fractions. Additional work will be needed to address these possibilities in further detail.

Our findings align with prior research. Recent work revealed that introduction of a missense mutation into *Tet1* in mESCs leads to a partial disruption of TET1–OGT interaction. This results in global DNA hypomethylation, consistent with the mutated TET1 protein detaching from TOPD complexes to trigger widespread DNA demethylation (Hrit et al. 2018). Similarly, a recent study observed global DNA demethylation and TE derepression after acute deletion of OGT in mESCs (Sepulveda et al. 2024). This strongly suggests that OGT loss disrupts TOPD complex stability, leading to the release of TET proteins and subsequent genome-wide DNA demethylation. Interestingly, the absence of TET enzymes themselves has been linked to widespread DNA demethylation, particularly in heterochromatin (López-Moyado et al. 2019). This phenomenon was proposed to stem from disruption of a shared protein complex important for both TET and DNMT enzyme recruitment. When TET enzymes are removed, this complex is disrupted, leading to the redistribution of DNMT enzymes across the genome (López-Moyado et al. 2019). Thus, in addition to the role of PROSER1 in restraining TET activity, it is interesting to speculate whether TOPD complexes may have additional roles, directly or indirectly, in regulating DNMT activity.

Pairwise TET interactions with specific partner proteins like OGT, PSPC1, and NONO have previously been identified (Chen et al. 2013; Deplus et al. 2013; Vella et al. 2013; Guallar et al. 2018; Li et al. 2020; Huang et al. 2022). However, our results imply that these interactions do not occur in isolation but can combine to form larger multimeric TOPD complexes that have functional roles in chromatin. Interestingly, mutations in several components of TOPD complexes beyond PROSER1 have been linked to neurodevelopmental disorders. These include X-linked variants of OGT (causing congenital disorder of glycosylation [OGT-CDG]) (Pravata et al. 2020; Authier et al. 2024) and NONO (causing NONO-associated syndromic disorder) (Mircsof et al. 2015; Reinstein et al. 2016; Roessler et al. 2023), as well as biallelic loss of TET3 (causing Beck–Fahrner syndrome) (Beck et al. 2020; Seyama et al. 2022), all of which display syndromic developmental delay, intellectual disability, and craniofacial dysmorphisms similar to features observed upon inactivation of PROSER1. Although mutations in TOPD components are likely to have additional pleiotropic effects, some common features across these disorders may stem from shared disruption of TOPD complexes during development.

Chromatinopathies represent an expanding category of congenital developmental disorders arising from disruptions in chromatin function and dysregulation of the epigenome (Nava and Arboleda 2024). Mutations within genes encoding critical epigenome regulators, encompassing both core components and accessory proteins such as PROSER1, contribute to this growing list of pathologies. In this study, we show that PROSER1 plays a central role in the assembly of multiprotein chromatin-associated TET complexes that shape the DNA methylome and support gene expression. Furthermore, mice lacking PROSER1 mirror the developmental defects seen in humans with homozygous PROSER1 loss-of-function mutations. We therefore propose that developmental syndromes caused by PROSER1 mutations should be designated as a novel form of chromatinopathy and that future investigations into TOPD-related developmental syndromes should leverage the growing understanding of TET enzyme function in development and disease. Our development of a PROSER1 knockout mouse model serves as both a system to gain insight into the underlying mechanisms and a preclinical model to explore the potential for therapeutic intervention in PROSER1-related developmental syndromes.

## Materials and methods

### PROSER1 knockout mouse line

To generate a mouse line with constitutive inactivation of PROSER1, fertilized 1 cell zygotes were injected with Cas9 mRNA and a small guide RNA (5′-GTGCTGGATGAAATTCGAA-3′) targeting exon 1 of *Proser1* (ENSMUSG00000049504). Founder animals harboring an allele with a disruptive 7 bp deletion starting at position 53,371,732 of chromosome 3 (build GRCm39) were selected for backcrossing to C57BL/6J animals and further breeding. All mouse studies were conducted in accordance with the regulations described in the UK Animals (Scientific Procedures) Act 1986 and approved by the Welfare and Ethical use of Animals Committee at the University of Dundee.

### mESCs

Mouse ESC lines were derived from blastocysts harvested from TET2^fl/fl^ animals (Moran-Crusio et al. 2011) and cultured feeder-free on gelatinized plates in “serum/2i/LIF” conditions (Hon et al. 2014) as follows: DMEM high-glucose plus GlutaMAX supplemented with 8.3% ESC-qualified fetal bovine serum (FBS; Gibco 16141079), 4.1% knockout serum replacement (Gibco 10828028), 1× penicillin/streptomycin (P/S), 0.1 mM β-mercaptoethanol (β-ME), 1× MEM nonessential amino acids (NEAA), 1× sodium pyruvate, 3 µM GSK3 inhibitor CHIR99021 (Sigma SML1046), 1 µM MEK1/2 inhibitor PD0325901 (Sigma PZ0162), and 100 ng/mL LIF-GST cleaved (DU1715; MRC Protein Phosphorylation and Ubiquitylation Unit [PPU] Reagents and Services Facility).

To generate PROSER1 KO mESCs, a guide RNA (gRNA) targeting exon 1 of mouse PROSER1 (5′-GTGCTGGATGAAATTCGAA-3′) was cloned into PX458 (Addgene plasmid 48138). After transfection into mESCs, GFP-positive cells were single-cell-sorted and genotyped by restriction fragment length polymorphism (RFLP) analysis using BstBI (NEB) digestion. Clones of interest were screened for loss of PROSER1 protein by Western blot, and indels were cloned and sequenced. Genotyping oligos are listed in Supplemental Table S2. To obtain isogenic TET2 knockout cells, TET2^fl/fl^ mESCs were transiently transfected with a pBABE-Cre plasmid and subcloned to identify multiple independent TET2 knockout lines. For re-expression of full-length PROSER1, the mouse *Proser1* coding sequence was amplified from cDNA using primers to add a C-terminal 2XFLAG tag and cloned into a Gateway entry vector (Thermo Scientific K240020SP). LR recombination with LR Clonase II enzyme mix (Invitrogen 10134992) was then performed to transfer the sequence-verified clone into a destination vector for PiggyBac transposition (PiggyBac-mProser1-FLAG-IRES-Blast, deposited at Addgene 226173). Cells were transfected with a 1:1 ratio of PiggyBac and pBase constructs using Lipofectamine 3000 (Invitrogen L3000008), and the pool of cells was selected with 5 µg/mL blasticidin (Sigma 203350) for ∼10 days prior to subcloning by limited dilution and isolation of clonal mESC lines.

For generation of mouse embryoid bodies (mEBs), mESCs were trypsinized and resuspended in mEB media as follows: DMEM high-glucose plus GlutaMAX, supplemented with 10% FBS (Scientific Laboratory Supplies [SLS]), 1× P/S, 0.1 mM β-ME, 1× NEAA, and 1× sodium pyruvate (all from Gibco). Cells were plated on low-attachment dishes (Sterilin Standard 90 mm Petri dishes [Thermo Fisher]) at 1.5 × 10^5^ cells/mL and cultured for 48 h at 37°C and 5% CO_2_ prior to harvest. For neuronal monolayer differentiation, mESCs were trypsinized and resuspended in N2B27 media as follows: 1:1 mix of advanced DMEM-F12 (12634010) and neurobasal (21103049) supplemented with 1× L-glutamine, 0.1 mM β-ME, 1× NEAA, 0.5× N-2 supplement (17502048), and 0.5× B-27 supplement (17504044) (all from Gibco). The cells were plated onto gelatin-coated plates at 1.0 × 10^5^ cells per 6 well plate. The media was changed every 2 days until harvest and analysis.

### Antibody generation

To generate a specific sheep polyclonal antibody against epitopes in the N terminus of PROSER1 (PROSER1-N), the coding sequence for mouse *Proser1* (amino acids 1–230) was cloned into pGex (GST-tagged) and pMex (MBP-tagged) vectors and used for *Escherichia coli* protein expression and purification. GST-PROSER1 (amino acids 1–230) was used for sheep antibody production, and bleeds were affinity-purified against MBP-PROSER1 (amino acids 1–230) to decrease unspecific reactivity and remove GST-specific antibodies. The PROSER1-N antibody is reactive against both mouse and human PROSER1. To generate a PROSER1-C-specific antibody (PROSER1-C), a 12 amino acid peptide [(C)SLQTGLSQSGWQ] corresponding to the C terminus of mouse PROSER1 was synthesized and coupled to KHL via the N-terminal cysteine. This conjugate was subsequently used for rabbit immunization, and bleeds were affinity-purified against immobilized (C)SLQTGLSQSGWQ peptide using SulfoLink immobilization kit for peptides (Thermo Scientific 44999). Sheep and rabbit immunization projects were performed in collaboration with the MRC PPU Reagents and Services Facility and Morovian Biotechnology Ltd., respectively.

### Immunoprecipitation and mass spectrometry (IP-MS)

Triplicates samples of wild-type and knockout mESC lines were harvested by trypsinization, counted, and lysed in zwitterionic lysis buffer supplemented with protease inhibitors as follows: 50 mM HEPES (pH 7.5), 150 mM NaCl, 10 mM NaF, 0.5% C_7_BzO (Sigma C0856), and 1× cOmplete protease inhibitors EDTA-free (Roche 11836170001). After 30 min of incubation on ice, the lysates were cleared by centrifugation at 20,000*g* for 30 min, and concentrations of supernatants were measured by Bradford assay and normalized. Immunoprecipitation was carried out overnight on ≥1 mg of protein in 1 mL of lysate at 4°C with rotation. PROSER1 IP-MS was carried out using PROSER1-C antibody (1 µg for 1 mL of 1.5 mg/mL lysate) (this study). TET2 IP-MS was carried out using TET2 antibody (Cell Signaling Technology D6C7K) at 0.5 µg for 1 mL of 1.5 mg/mL lysate. TET2 IP-MS was initially performed on cells cross-linked for 45 min with 2 mM disuccinimidyl glutarate (DSG; Thermo Scientific 20593) at room temperature, but subsequent TET2 IP and Western blot analysis of noncross-linked lysates confirmed that the observed TET2–TOPD interactions were not dependent on protein–protein cross-links. Thirty microliters of protein G magnetic beads (Invitrogen 10003D) per 1 mL of lysate was used to bind the antibody and was incubated with the lysates for 3 h at 4°C with rotation. The beads were washed five times in ice-cold wash buffer (10 mM Tris-HCl at pH 8, 1 mM EDTA, 150 mM NaCl, 1× cOmplete protease inhibitors EDTA-free). Proteins were eluted by resuspension in 2× LDS sample buffer and incubation with shaking at 1500 rpm for 15 min at 70°C prior to addition of 10 mM DTT and 50 mM iodoacetamide for reduction and alkylation. For mass spectrometry analysis of DSG-cross-linked immunoprecipitation samples (TET2 IP-MS), the reduced and alkylated eluates were purified by SDS-PAGE and subjected to in-gel tryptic digestion prior to desalting (C18 column) and 6-plex TMT labeling using TMTsixplex isobaric label reagent set (Thermo Scientific) according to the manufacturer's instructions. The pooled and desalted samples were run on an Orbitrap Fusion Lumos Tribrid spectrometer (Thermo Fisher) with SPS-MS3 workflow. For mass spectrometry analysis of uncross-linked PROSER1-C immunoprecipitation samples, the reduced and alkylated eluates were purified by SDS-PAGE and subjected to in-gel tryptic digestion as described above. Eluted peptides were analyzed on an Orbitrap Exploris 480 mass spectrometer (Thermo Scientific) using a 120 min gradient elution and data-independent acquisition.

### ChIP-seq

Biological replicate samples of wild-type and knockout mESC lines were trypsinized and double-cross-linked (DSG+FA) by resuspension in ice-cold PBS supplemented with 2 mM DSG (Thermo Scientific 20593) and incubation for 30 min at room temperature, followed by addition of formaldehyde (Thermo Scientific 28906) to a final concentration of 1% and a further 10 min of incubation at room temperature. Quenching was performed by addition of glycine to a final concentration of 125 mM. Cells were washed with PBS and lysed by resuspension in SDS buffer (50 mM Tris-HCl at pH 8.1, 100 mM NaCl, 5 mM EDTA, 0.5% SDS supplemented with 1 mM phenylmethylsulfonyl fluoride [PSMF]). Chromatin was collected by centrifugation at 300*g* for 6 min at 20°C and resuspended in IP buffer (50 mM Tris-HCl at pH 8.6, 100 mM NaCl, 5 mM EDTA, 1.6% Triton X-100, 0.3% SDS). Sonication was carried out using a Bioruptor Pico (Diagenode), and fragmentation conditions (to obtain 100–500 bp fragments) were optimized for each experiment before continuing. Sonicated samples were cleared by centrifugation at 20,000*g* for 20 min at 4°C, and the pellet was discarded. Protein concentration was estimated by Bradford assay (Bio-Rad 500-0006), and the concentrations were normalized across samples. SDS-free buffer (50 mM Tris-HCl at pH 8.1, 100 mM NaCl, 5 mM EDTA) was used to dilute SDS in samples to a final concentration of 1%, and the samples were precleared by 3 h of incubation with Protein G Sepharose beads (Cytiva GE17-0618-01).

Immunoprecipitation was carried out overnight on 600 µg of protein (for PROSER1 and TET2) or 60 µg of protein (for H3K4me1 andH3K27ac) in 1 mL of buffer. Antibody quantities were as follows: for PROSER1, PROSER1-N antibody (this study) (sheep polyclonal), 1.2 µg of antibody to 600 µg of chromatin; for TET2, TET2-N antibody (Rasmussen et al. 2019) (rabbit polyclonal), 1 µg of antibody for 600 µg of chromatin; for H3K4me1 (Cell Signaling Technology 5326) (rabbit monoclonal), 10 µL of antibody to 60 µg of chromatin); and for H3K27ac (Thermo Fisher MA5-23516) (mouse monoclonal), 4 µg of antibody to 60 µg of chromatin). Antibody–DNA complexes were immunoprecipitated by 3 h of incubation with 30 µL of 50% slurry of Protein G Sepharose beads (Cytiva GE17-0618-01) and washed three times with low-salt wash buffer (20 mM Tris-HCl at pH 8.0, 150 mM NaCl, 2 mM EDTA, 0.1% SDS, 1% Triton X-100), twice with high-salt wash buffer (20 mM Tris-HCl at pH 8.0, 500 mM NaCl, 2 mM EDTA, 0.1% SDS, 1% Triton X-100), and once with IP buffer with a final concentration of 0.1% SDS. Beads were then resuspended in decross-linking buffer (1% SDS, 100 mM NaHCO_3_) and incubated with shaking at 1200 rpm overnight at 65°C. DNA was purified using the Monarch PCR and DNA cleanup kit. Libraries were prepared from 1–3 ng of purified DNA input using TruSeq adapters (Illumina) and sequenced on a NovaSeq 6000 system with 150 bp paired-end sequencing.

See the Supplemental Material for further information about the materials and methods used in this study.

### Data availability

Raw and processed data sets are available for download at the Gene Expression Omnibus (GEO) database under accession number GSE273517.

## Supplemental Material

Supplement 1

Supplement 2
