# Supplementary material for: PROSER1 modulates DNA demethylation through dual mechanisms to prevent syndromic developmental malformations
Source: Genes Dev. 2024 Nov-Dec;38(21-24):952–64. doi: 10.1101/gad.352176.124 (PMC11610933; doi:10.1101/gad.352176.124)
Supplement: Supplement 1 [file Supplemental_Figures_and_Methods.pdf]

Supplemental information

PROSER1 Modulates DNA Demethylation through Dual Mechanisms to Prevent Syndromic Developmental Malformations

Anna Fleming[1], Elena V. Knatko[1], Xiang Li[1], Ansgar Zoch[2,3,8], Zoe Heckhausen[4,5], Stephanie Stransky[6], Alejandro J. Brenes[7], Simone Sidoli[6], Petra Hajkova[4,5], Dónal O'Carroll[2,3], Kasper D. Rasmussen[1]

[1]Division of Molecular, Cellular, and Developmental Biology, University of Dundee, Dundee, UK
[2]Centre for Regenerative Medicine, Institute for Regeneration and Repair, Institute for Stem Cell Research, University of Edinburgh, 5 Little France Drive, Edinburgh, EH16 4UU, UK.
[3]Wellcome Centre for Cell Biology, University of Edinburgh, Michael Swann Building, Max Born Crescent, Edinburgh, EH9 3BF, UK.
[4]MRC Laboratory of Medical Sciences, London, UK
[5]Institute of Clinical Sciences, Faculty of Medicine, Imperial College London, London, UK
[6]Department of Biochemistry, Albert Einstein College of Medicine, Bronx, NY, USA
[7]Division of Cell Signalling and Immunology, University of Dundee, Dundee, UK
[8]present address: MRC Human Genetics Unit, Institute of Genetics and Cancer, University of Edinburgh, Western General Hospital, Crewe Road South, Edinburgh EH4 2XU, Edinburgh, UK

Corresponding author: kdrasmussen@dundee.ac.uk

CONTENT

Supplemental Figure 1
Supplemental Figure 2
Supplemental Figure 3
Supplemental Figure 4
Supplemental Figure 5
SUPPLEMENTAL METHODS
SUPPLEMENTAL REFERENCES

Fleming_Fig S1

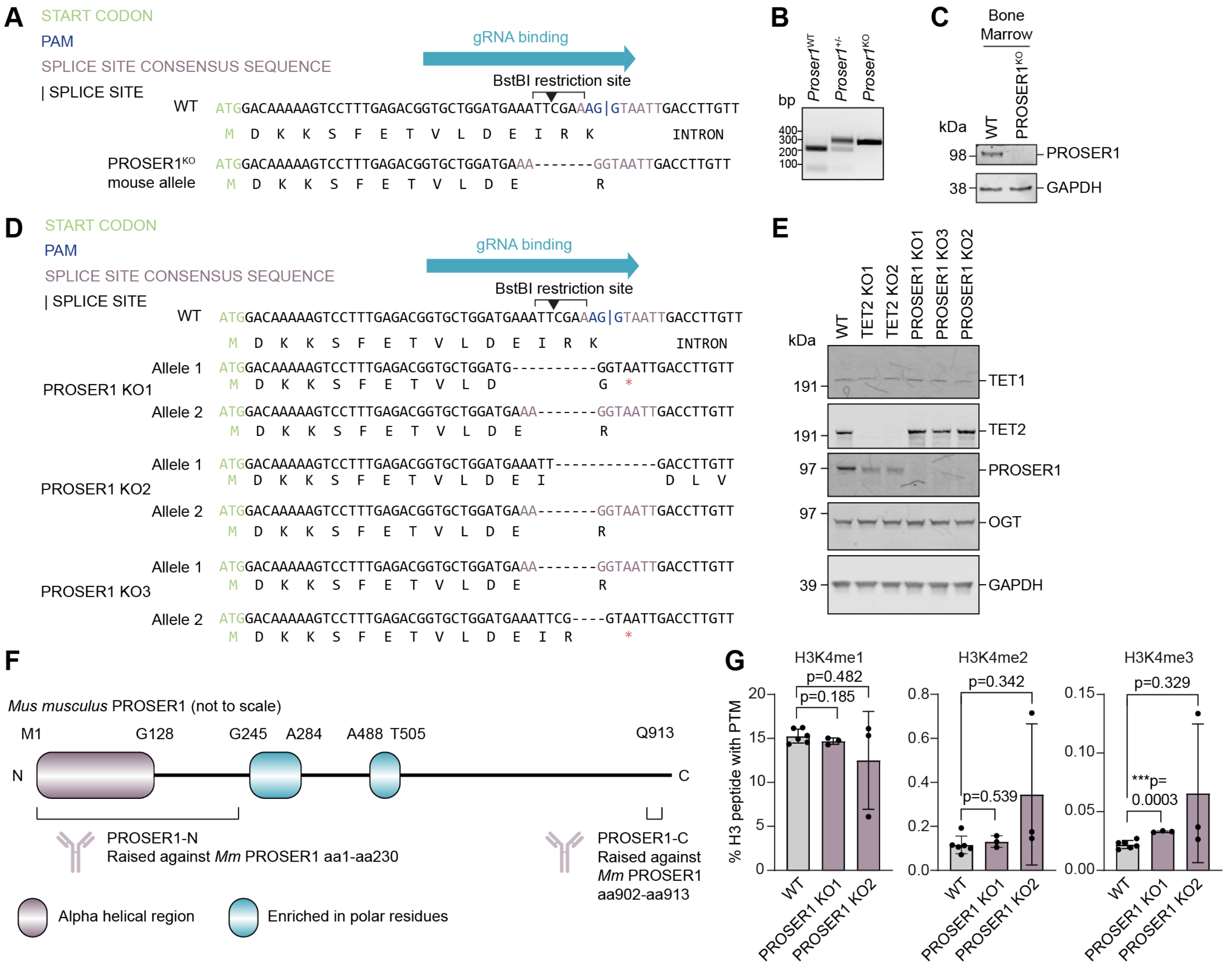

Supplemental Fig. S1 [A] DNA and amino acid sequences of the region surrounding the PROSER1 gRNA binding site in WT and PROSER1^{KO} mice. Green text indicates the start codon, blue the PAM sequence, purple the splice-site consensus sequence. The BstBI restriction site is indicated above the DNA sequence. PROSER1^{KO} mice contains a 7bp deletion that disrupts the reading frame and introduces a premature stop codon. [B] Representative agarose gel showing genotyping of a wildtype (WT), PROSER1 heterozygous (+/-), and knockout (KO) mouse. bp indicates base pairs [C] Western blot of lysates from the bone marrow of a WT or PROSER1^{KO} mouse. GAPDH was probed as a loading control. [D] As in A, but in three clonal PROSER1 KO mESC lines. When an allele has no purple regions this indicates that the splice-site consensus sequence is lost. Premature stop codons are indicated with red asterisks. Both alleles in these three clones either had a frameshift mutation or lost the splice-site consensus sequence. [E] Western blot of lysates from WT, TET2 KO or PROSER1 KO clonal mESCs. GAPDH was probed as a loading control. [F] Illustration of *Mus musculus* PROSER1 (not to scale), with the binding regions of our PROSER1-N and PROSER1-C antibodies indicated below. The single letter

amino acid codes and the residue numbers at which the indicated regions begin and end are given above. [G] Global enrichment of H3K4me1, 2 or 3 in WT and PROSER1 KO1 and 2 mESCs as measured by quantitative MS. Error bars represent the mean ±SD. Statistical significance was measured by unpaired two-tailed *t*-test with Welch's correction.

Fleming_Fig S2

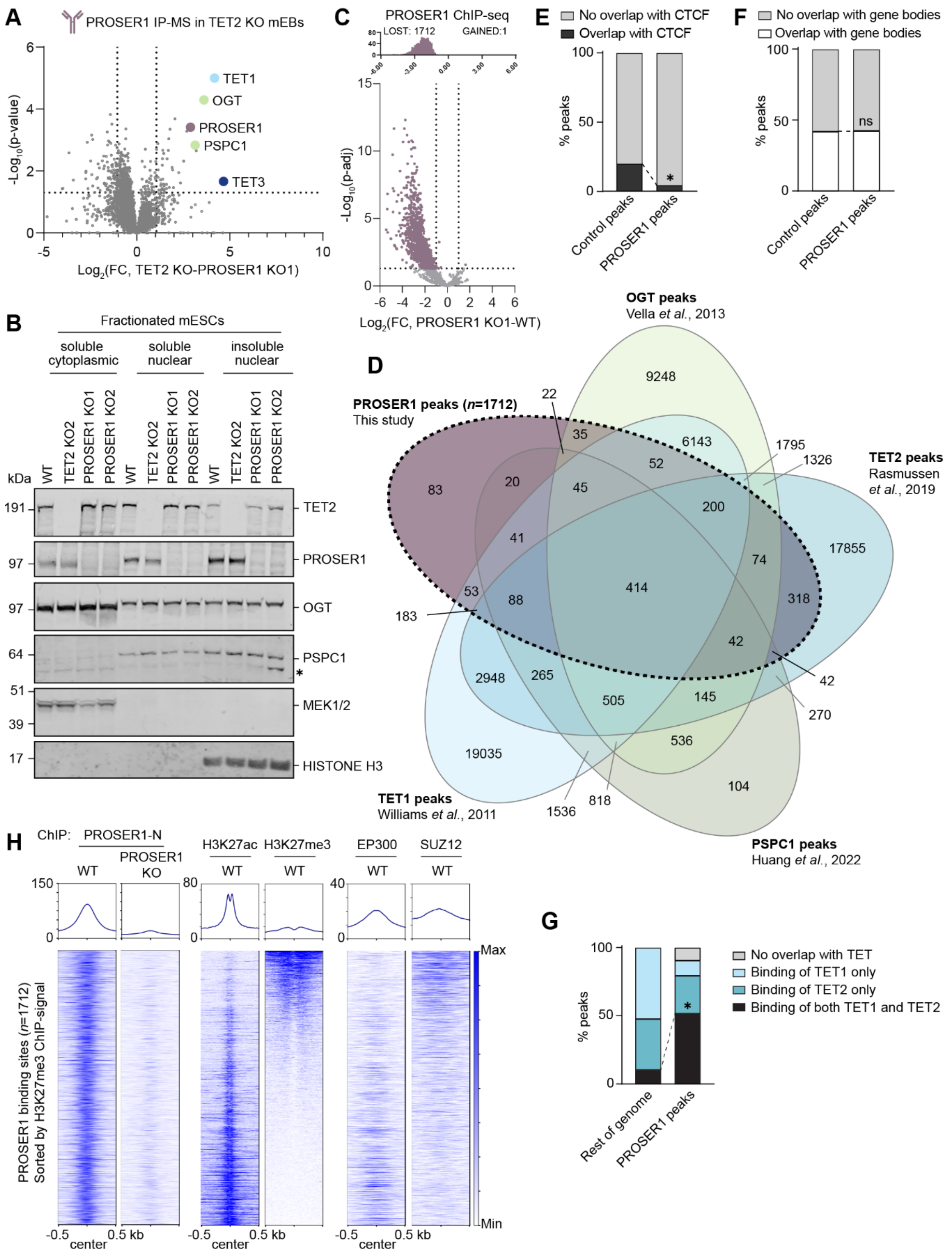

Supplemental Fig. S2 [A] Volcano plot showing protein hits from α-PROSER1-C immunoprecipitation and mass spectrometry (IP-MS) in TET2 KO mEBs (n=3 biological replicates). Protein enrichment is compared to parallel IP-MS in PROSER1 KO mEBs. Dotted lines indicate 2-fold change and p-value 0.05.

Proteins of interest are highlighted and identified. See Supplemental Tab. S5 for full list of enriched proteins. [B] Western blot of fractionated WT, TET2 KO and PROSER1 KO1 and 2 mESCs. MEK1/2 and histone H3 were run as fractionation controls for the cytoplasmic and chromatin fractions, respectively. * indicates a non-specific reactive band. [C] Volcano plot and frequency histogram showing changes in PROSER1-chromatin binding identified by PROSER1-N ChIP-seq in WT and PROSER1 KO1 mESCs (n=2 biological replicates). Dotted lines indicate 2-fold change and p-adj 0.05. PROSER1-chromatin binding sites where p-adj<0.05 and abs(fold change≥2) are highlighted in purple and their number given. [D] Venn diagram showing overlap between PROSER1, OGT, TET2, PSPC1 and TET1 peaks in mESCs. The number of overlapping peaks in each combination of peaksets is indicated. All peaksets but PROSER1-N peaks were generated from publicly available data (Huang et al. 2022; Rasmussen et al. 2019; Vella et al. 2013; Williams et al. 2011). [E] Percentage of PROSER1-N peaks or matched controls that overlap with CTCF binding. CTCF binding sites were obtained from publicly available data (Song et al. 2022). * p<0.0001, two-tailed Fisher's exact test. [F] As E, but overlapping gene bodies. [G] Percentages of genomic regions from TET1 (Williams et al. 2011) and TET2 (Rasmussen et al. 2019) peaksets that are associated with TET1 or TET2 binding, or both, within PROSER1-N peaks or the rest of the genome. * p<0.0001, two-tailed Fisher's exact test. [H] Heatmaps and mean values of normalized ChIP-seq signals for PROSER1-N and activating (P300 and H3K27ac) and repressive (H3K27me3 and SUZ12) chromatin features centered at 1712 high-confidence PROSER1 binding sites in mESCs. P300 and SUZ12 enrichment were generated from (Wang et al. 2017) and (Højfeldt et al. 2018) respectively, and regions were ranked based on the H3K27me3 ChIP-seq signal. Heatmaps were generated using DeepTools software (Ramírez et al. 2014).

Fleming_FigS3

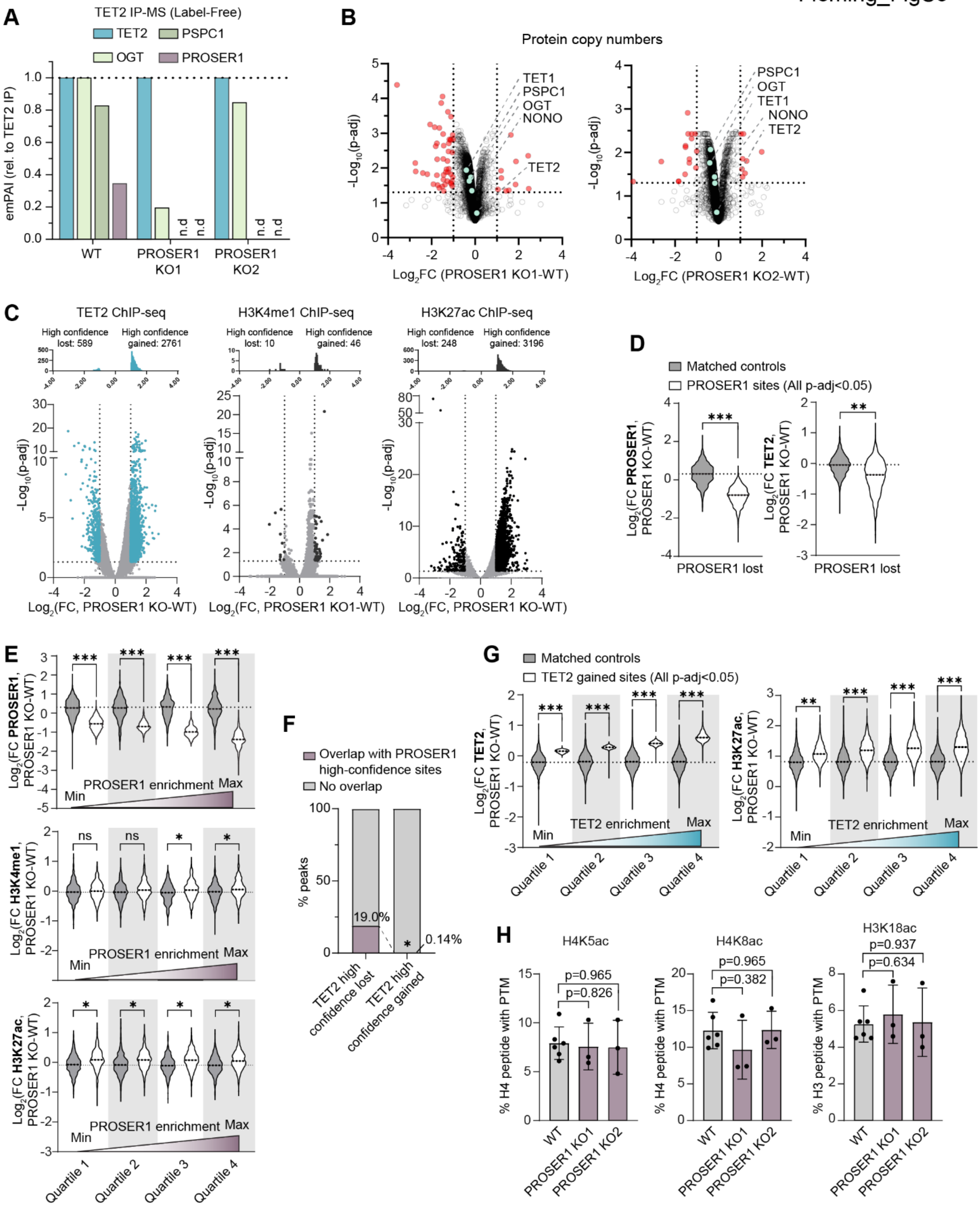

Supplemental Fig. S3 [A] Estimated protein abundance indicated by exponentially modified protein abundance index (emPAI) of TET2, OGT, PSPC1 and PROSER1 in TET2 IP-MS normalized to TET2 IP efficiencies in each experiment (*n*=1 biological replicate). n.d., not detected. [B] Volcano plots showing protein copy numbers determined by whole-proteome MS on PROSER1 KO1 and KO2 mESCs (*n*=3

biological replicates). Protein enrichment is compared to parallel MS in WT mESCs. Dotted lines indicate 2-fold change and p-adj 0.05. Components of the TOPD complexes are highlighted and identified [C] Volcano plot and frequency histograms showing changes in TET2 (left), H3K4me1 (center) or H3K27ac (right) enrichment on chromatin identified by ChIP-seq in WT and PROSER1 KO1 mESCs (*n*=3 biological replicates). Dotted lines indicate 2-fold change and p-adj 0.05. Sites where p-adj<0.05 and Abs(fold change≥2) are highlighted in blue or black (for TET2 or H3K4me1 and H3K27ac, respectively) and their number is given. [D] Fold change in PROSER1 binding (left) or TET2 binding (right) based on normalized read counts within PROSER1 DOWN and PROSER1 UP sites (p-adj<0.05) (white) or matched control regions (gray) upon PROSER1 loss. The effect sizes of binding loss compared to matched control regions were measured with Cohen's d. * d>0.3 (small effect), ** d>0.6 (medium effect), *** d>0.9 (large effect). ns, not-significant. [E] Fold change in PROSER1 binding (above) or enrichment of H3K4me1 (center) or H3K27ac (below) within PROSER1 peaks or matched control regions upon PROSER1 loss. PROSER1 peaks were sorted by Log_2(fold change in PROSER1, KO-WT) and divided into equal-sized quartiles (white). Controls (gray) were generated for each quartile. The effect sizes of binding loss compared to matched control regions were measured with Cohen's d. * d>0.3 (small effect), ** d>0.6 (medium effect), *** d>0.9 (large effect). ns, non-significant. [F] Percentage of PROSER1 peaks that overlap with high-confidence (p-adj<0.05, Abs(fold change≥2)) sites with gain or loss of TET2 binding. * p<0.0001, two-tailed Fisher's exact test. [G] As D, but fold change in TET2 binding (left) or H3K27ac enrichment (right) at quartiles of all sites with significant (p-adj<0.05) gain of TET2 binding. [H] Global enrichment of the p300 acetylation sites H4K5ac (left), H4K8ac (center) and H3K18ac (right) in WT and PROSER1 KO1 and 2 mESCs as measured by quantitative MS. *n*=3 biological replicates of PROSER1 KO lines and 6 of WT. Error bars represent the mean ±SD. Statistical significance was measured by unpaired two-tailed *t*-test with Welch's correction.

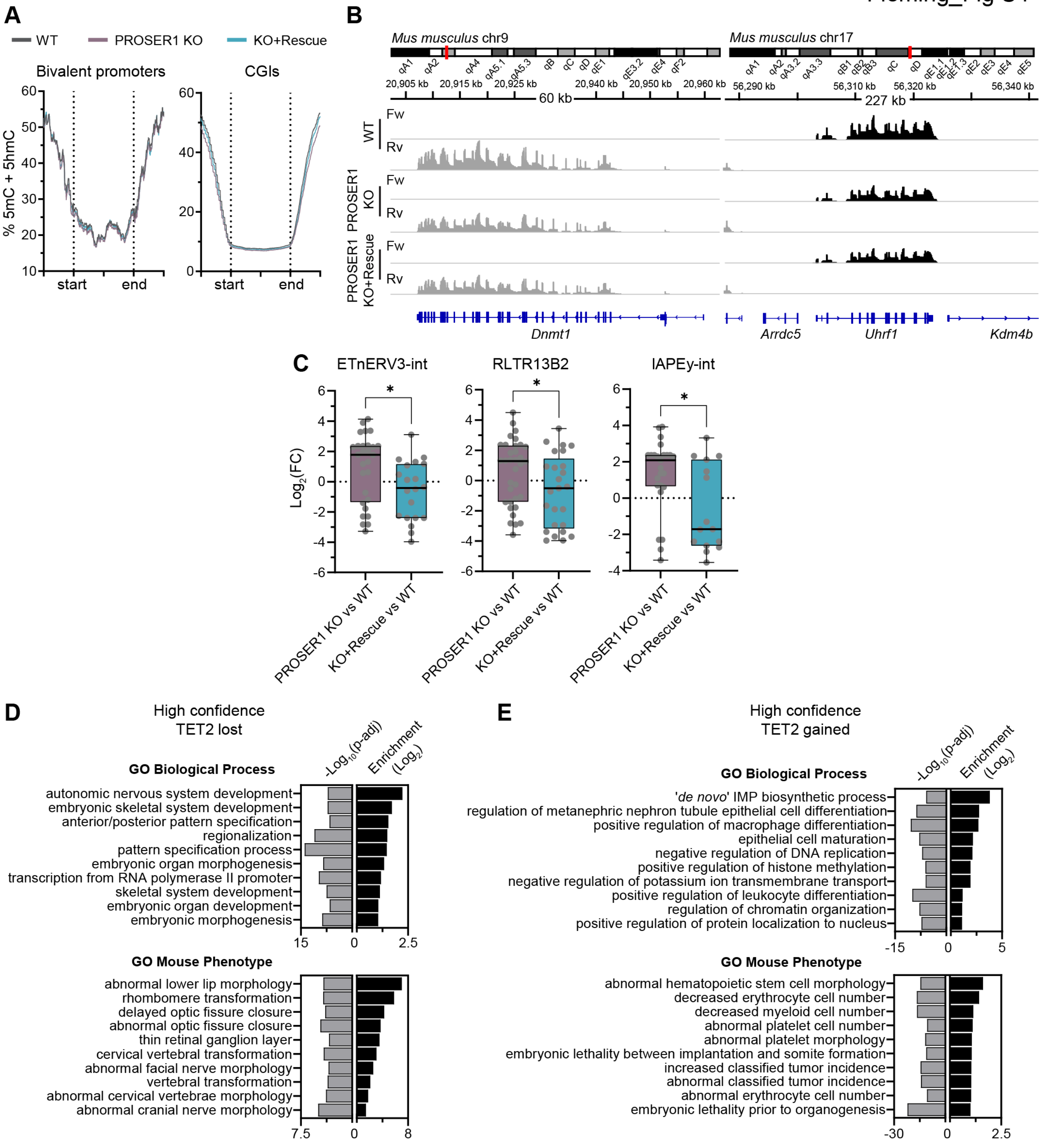

Supplemental Fig. S4 [A] Quantitation trend plots (window size 50 bp, step 50 bp, 1 kb flanking sequence) of DNA methylation quantified by EM-seq in bivalent promoters and CGIs for CpG sites covered by minimum 10 EM-seq reads in all samples in WT, PROSER1 KO and PROSER1 KO+Rescue mESCs. [B] Coverage of forward (Fw) or reverse (Rv) transcripts from RNA-seq (n=2 biological replicates) in WT, PROSER1 KO and KO+Rescue mESCs at the regions surrounding *Dnmt1* (left) and *Uhrf1* (right). [C] Box plots showing *TEtranscripts* differential expression analysis of TEs. Dots represent expression of individual TEs whose expression was quantified using only uniquely mapped RNA-seq

reads. The box plots compare differential expression of TEs in PROSER1 KO vs WT and PROSER1 KO+Rescue vs WT for ETnERV3-int (left), RLTR13B2 (middle), and IAPEy-int (right) families of endogenous retroviral elements, all found to be significantly deregulated upon PROSER1 KO. Statistical significance was measured by unpaired two-tailed *t*-test with Welch's correction (* $p<0.05$). [D] Bar charts showing enriched gene ontology (GO) terms identified by GREAT (McLean et al. 2010) for genes with a regulatory domain overlapping high-confidence sites with loss of TET2 binding upon PROSER1 KO. [E] same as [D], but for sites with increased TET2 binding upon knockout of PROSER1.

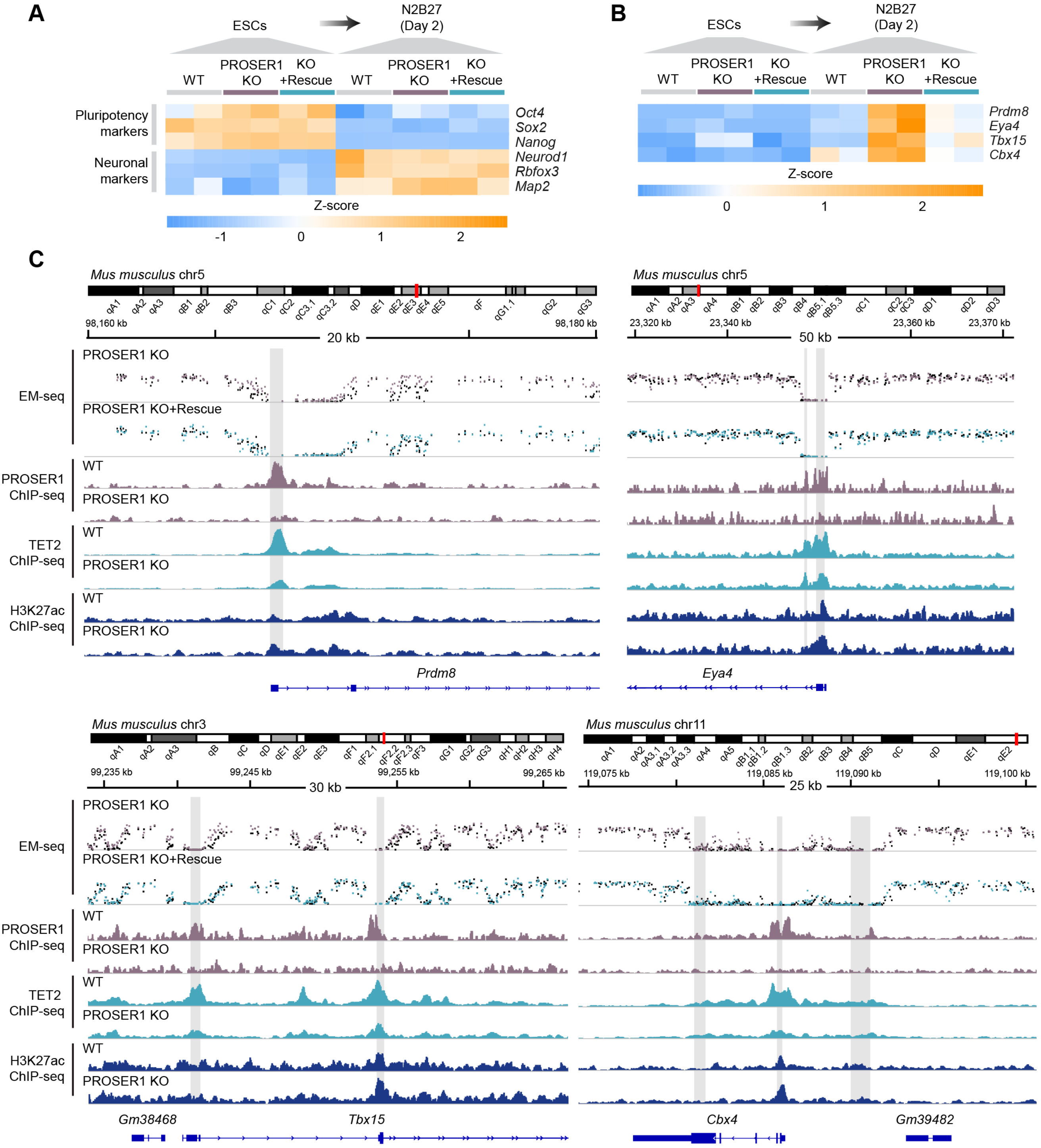

Supplemental Fig. S5 [A] Heatmap showing the changes in expression (Z-score of normalized read counts from RNA-seq) of key pluripotency markers (above), and neuronal markers (below) in WT, PROSER1 KO and PROSER1 KO+Rescue mESCs (left) or cells subjected to monolayer differentiation in N2B27 medium (right). Gene symbols for each row in the heatmap are given on the right of the plot. [B] As in A, but for selected developmental genes co-bound by PROSER1 and TET2 whose expression correlated with PROSER1 expression. [C] Tracks showing the region surrounding the developmental

genes *Prdm8*, *Eya4*, *Tbx15*, and *Cbx4*. Data in the top two panels represent pooled EM-seq methylation calls. Data from WT mESCs are shown in black and data from PROSER1 KO and PROSER1 KO+Rescue are overlaid in purple or blue, respectively. Below, tracks represent ChIP-seq coverage in PROSER1 ChIP-seq (*n*=2 biological replicates), and TET2 or H3K27ac ChIP-seq (*n*=3 biological replicates). CGIs are indicated in gray.

Supplemental Methods

Generation of PROSER1 knockout mouse line and animal husbandry

To generate a mouse line with constitutive inactivation of PROSER1, fertilised 1-cell zygotes were injected with Cas9 mRNA and a small guide RNA (5'- GTGCTGGATGAAATTCGAA-3') targeting exon 1 of *Proser1* (ENSMUSG00000049504). Injected zygotes were cultured to blastocyst stage and implanted into pseudo-pregnant females. Genomic DNA harvested from offspring was screened for the presence of out-of-frame indels. Founder animals harboring an allele with a disruptive 7 bp deletion starting at position 53,371,732 of chromosome 3 (Build GRCm39) were selected for backcrossing to C57BL/6J animals and further breeding.

Genotyping was performed as follows: genomic DNA was extracted by incubating ear notches in 75 µL Lysis buffer (25mM NaOH, 0.2mM EDTA, pH12.0) at 98°C for 30 min, cooling briefly on ice and neutralising by the addition of 75 µL of 40 mM Tris-HCl, pH 5.0. Genotyping PCR was performed using *Proser1* RFLP primers (see Supplemental Tab. S2), and Phire Hot Start II PCR Master Mix (Thermo) according to the manufacturer's instructions. The PCR product was then digested with BstBI (NEB) and resolved on a 2% agarose gel.

All mouse studies were conducted in accordance with the regulations described in the UK Animals (Scientific Procedures) Act 1986 and approved by the Welfare and Ethical use of Animals Committee of the University of Dundee. Experimental design was in line with the 3Rs principles of replacement, reduction, and refinement (www.nc3rs.org.uk). Mice were bred and maintained at the SLS-WTB Resource Unit of the University of Dundee, with free access to water and food (pelleted RM1 diet from SDS Ltd., Witham, Essex, UK), on a 12 hr light/12 hr dark cycle, 35% humidity. For microCT scanning, $PROSER1^{WT}$ and $PROSER1^{KO}$ littermates were euthanized and fixed in 10% formalin and preserved at 4°C prior to imaging. The heads were positioned and imaged in the scanner bed of a nanoPET/CT scanner (Mediso, Hungary), and volumetric and morphological analysis performed using PMOD software (https://www.pmod.com/web/).

mESCs

Mouse ESC lines were derived from blastocysts harvested from $TET2^{fl/fl}$ animals and cultured feeder-free on gelatinized plates in 'Serum/2i/Lif' conditions (Hon et al. 2014): DMEM high-glucose plus GlutaMAX, supplemented with 8.3% ES-certified foetal bovine serum (ES-FBS), 4.1% Knock Out Serum Replacement (KOSR), 1x Pen/Strep (P/S), 0.1 mM β-mercaptoethanol (β-ME), 1x MEM non-essential amino acids (NEAA), 1x sodium pyruvate (all supplied by Gibco), 3 µM GSK3 inhibitor (Sigma, CHIR99021), 1 µM MEK1 inhibitor (Sigma, PD0325901), and 100 ng/mL LIF-GST cleaved (DU1715, MRC

PPU Reagents and Services facility). Cells were plated at $0.02x10^6$ cells/mL and incubated at 37°C and 5% CO_2. To obtain isogenic TET2 knockout cells, $TET2^{fl/fl}$ mESCs were transiently transfected with a pBABE-Cre plasmid and subcloned to identify multiple independent TET2 knockout lines.

To generate PROSER1 KO cells, mESCs were transfected with pSpCas9(BB)-2A-GFP containing a guide RNA (gRNA) targeting exon 1 of mouse PROSER1 (ENSMUSG00000049504) (see Supplemental Tab. S2) and single-cell sorted (SONY SH800S) by GFP expression. Single-cell sorted clones were expanded under normal mESC culture conditions until genomic DNA or protein could be extracted. Genomic DNA was extracted and as the gRNA cut site overlapped a BstBI restriction site, initial clone screening was carried out by restriction fragment length polymorphism (RFLP) analysis. The region of interest was amplified by PCR using *Proser1* RFLP primers (see Supplemental Tab. S2) and Q5 polymerase, then purified using the Monarch PCR and DNA Cleanup Kit (NEB). 200 ng of the purified PCR product was subjected to digestion by BstBI (NEB) for 1 hr at 37°C. The resulting fragments were analysed by agarose gel electrophoresis, with an uncut product representing a clone with homozygous indels. Clones which appeared to contain indels within the region of interest were then screened for loss of PROSER1 protein by Western blot. Furthermore, once clones had been selected the region of interest was cloned out and sequenced. Briefly, the region of interest was amplified by PCR using Q5 polymerase (primers as above for RFLP analysis), then purified using the Monarch PCR and DNA Cleanup Kit (NEB). This product was ligated into pSC-B-AMP/KAN vector using the StrataClone Blunt PCR Cloning Kit (Agilent) with incubation overnight at 16°C. Following ligation, the plasmid was transformed into chemically competent DH5-α and selected with blue-white screening. Plasmids were sequenced by Sanger sequencing.

To generate PROSER1 KO+Rescue cells, the mouse *Proser1* coding sequence was amplified from cDNA using primers which added a C-terminal FLAG tag (see Supplemental Tab. S2), purified, and cloned into an entry vector for the Gateway cloning system (pENTR/D-TOPO, Invitrogen). LR recombination was performed to clone PROSER1-FLAG into a PiggyBAC-GW-IRES-Blast destination vector with blasticidin resistance using Gateway LR Clonase II Enzyme mix (Invitrogen). Plasmids were amplified and sequenced, and PiggyBAC-Proser1-Flag-IRES-Blast as well as pBase plasmids were transfected into PROSER1 KO mESCs by Lipofectamine 3000 transfection (Invitrogen). Transfected cells were selected by treatment with Blasticidin-S at 5 µg/mL (Merck).

Differentiation of mESCs into mEBs

mESCs were trypsinised and resuspended in embryoid body (EB) media: DMEM, high glucose, GlutaMAX Supplement, supplemented with 10% Fetal Bovine Serum for Cell Culture (FBS) (Scientific Laboratory Supplies (SLS)), 100 IU/mL P-S, 1X Sodium Pyruvate, 1X NEAA, 0.1 mM β-ME. Cells were

plated on low attachment dishes (Sterilin Standard 90 mm Petri Dishes (Thermofisher Scientific)) at $0.15x10^6$ cells/mL and cultured at 37°C, 5% CO_2 for 48 hr prior to harvest.

Fractionation of mESCs

Cells were harvested and washed twice in ice-cold PBS before being resuspended in fractionation buffer A supplemented with protease inhibitors: 10 mM HEPES pH 7.9, 10 mM KCl, 1.5 mM $MgCl_2$, 340 mM sucrose, 10% glycerol, 1 mM DTT, 0.1% Triton X-100, 1X cOmplete protease inhibitors, EDTA free (Roche) and incubated on ice for 8 min. The lysates were centrifuged at 1300 xg and 4°C for 4 min and the supernatant (cytoplasmic fraction) separated from the pellet (nuclear fraction). The pellet was washed once in fractionation buffer A supplemented with protease inhibitors and the nuclei lysed by addition of fractionation buffer B supplemented with protease inhibitors: 3 mM EDTA, 0.2 mM EGTA, 1 mM DTT, 1X cOmplete protease inhibitors, EDTA free. Extraction was performed by incubation on ice for 30 min, during which time the cytoplasmic fraction was clarified by high-speed centrifugation at 20 000 xg and 4°C for 15 min and the supernatant retained. The nuclear lysate was further separated into soluble and insoluble fractions by centrifugation at 1700 xg and 4°C for 5 min. The resulting insoluble pellet was washed once in fractionation buffer B with protease inhibitors and resuspended in 2X LDS sample buffer (Thermo) and supplemented with 0.1 M $MgCl_2$ before being digested by Benzonase (Merck) for 1 hr at room temperature.

Antibody generation

To generate a specific sheep polyclonal antibody against epitopes in the N-terminus of PROSER1 (PROSER1-N), the coding sequence for mouse *Proser1* (aa1-aa230) was cloned into pGex (GST-tagged) and pMex (MBP-tagged) vectors and used for *E.coli* protein expression and purification. GST-PROSER1 (aa1-230) was used for sheep antibody production and bleeds were affinity-purified against MBP-PROSER1 (aa1-230) to decrease unspecific reactivity and remove GST-specific antibodies. To generate a PROSER1-C specific antibody (PROSER1-C), a 12aa peptide ((C)SLQTGLSQSGWQ) corresponding to the C-terminus of mouse PROSER1 was synthesized and coupled to KHL via the N-terminal cysteine. This conjugate was subsequently used for rabbit immunisation and bleeds were affinity purified against immobilised (C)SLQTGLSQSGWQ peptide using SulfoLink® Immobilization Kit for Peptides (ThermoFisher). Sheep and Rabbit immunisation projects were performed in collaboration with MRC PPU Reagents and Services facility and Morovian Biotechnology Ltd, respectively.

Immunoprecipitation

For crosslinked IP (TET2-N IP-MS), mESCs were harvested by trypsinisation, counted and crosslinked by the addition of 2 mM disuccinimidyl glutarate (Thermo) in ice-cold PBS and incubation at room temperature for 45 min with rolling. Crosslinking was quenched by the addition of glycine (Sigma) to a final concentration of 125 mM. For both crosslinked IP (TET2-N IP-MS) and native IP (PROSER1-C IP, TET2 IP-WB), cell lysates were prepared by 30 min incubation on ice in zwitterionic lysis buffer supplemented with protease inhibitors: 50 mM HEPES pH 7.5, 150 mM NaCl, 10 mM NaF, 0.5% C_7BzO (Sigma), 1X cOmplete protease inhibitors, EDTA free and 30 min centrifugation at 20 000 xg. Concentrations were measured by Bradford assay and normalised. Immunoprecipitation was carried out overnight on ≥1 mg protein in 1 mL lysate (see Supplemental Tab. S1 for antibody dilutions) and at 4°C with rotation. 30 µL protein G magnetic beads (Invitrogen) per 1 mL lysate were used to bind the antibody and were incubated with the lysates for 3 hr at 4°C with rotation. The beads were washed 5X in ice-cold wash buffer supplemented with 1X protease inhibitors: 10 mM Tris-HCl pH 8, 1 mM EDTA, 150 mM NaCl, 1X cOmplete protease inhibitors, EDTA free. Proteins were eluted by resuspension in 2X LDS sample buffer and incubation at 70°C with shaking at 1500 RPM for 15 min. For analysis by Western blotting, samples were supplemented with DTT to a final concentration of 0.1 M and boiled for 10 min. For analysis by mass spectroscopy, samples were reduced and alkylated by addition of 10 mM DTT and 50mM Iodoacetamide, respectively.

Western Blotting

Cell lysates were prepared as described in Immunoprecipitation. Between 5 and 50 µg protein per well was subjected to SDS-PAGE on a Bis-Tris or Tris-Acetate gel (ThermoFisher). Proteins were transferred to nitrocellulose membrane (Amersham) and probed using primary antibodies against proteins of interest as described in Supplemental Tab. S1. Imaging was carried out using photographic film or LI-COR system (LI-COR Biosciences). Secondary antibodies used are specified in Supplemental Tab. S1.

Whole proteome mass spectrometry

Whole cell lysates were processed for S-Trap™ peptide purification according to manufacturer's instructions (ProtiFi). Cells were harvested and washed twice in ice-cold PBS before being resuspended in SDS lysis buffer: 50 mM TEAB pH 8.5, 5% SDS and sonicated for 10 cycles of 30 sec on 30 sec off using a Bioruptor Pico (Diagenode). Lysates were clarified by centrifugation at 13 000 xg, 4°C for 8 min and the supernatant retained. The protein concentration was normalised using BCA protein assay (ThermoFisher). DTT was added to a final concentration of 20 mM and the lysates boiled for 10 min then cooled to room temperature. Freshly dissolved iodoacetamide was added to a final concentration of 40 mM and incubated in the dark at room temperature for 30 min. Samples were centrifuged at 13

000 xg for 8 min and the supernatant retained. 12% (v/v) phosphoric acid was added to the samples at a volume:volume ratio of roughly 1:10 acid:supernatant, followed by 7X the original lysis volume of S-trap binding buffer: 100 mM TEAB pH 7.55, 90% aqueous methanol. Samples were loaded onto S-trap mini columns and the columns washed four times by addition of S-trap binding buffer and centrifugation. MS-grade trypsin (Pierce) was resuspended in 50 mM TEAB and loaded onto the columns so that the final ratio of trypsin:protein was approximately 1:40 and incubated overnight at 37°C. Fresh trypsin was added to the columns and incubated for a further 4 hr at 37°C. The digested peptides were eluted by addition of 50 mM TEAB to the column, followed by 0.2% aqueous formic acid and finally 50% aqueous acetonitrile with 0.2% formic acid. The eluates were pooled and dried down before being resuspended in 1% formic acid. A defined amount (1.5 µg) of each sample was quantified using a 120 min gradient elution and data-independent acquisition (DIA) on an Orbitrap Exploris 480 Mass Spectrometer (ThermoFisher).

Whole proteome data analysis

The raw files were processed using Spectronaut® software (Bruderer et al. 2015) (Biognosys) and searched against mouse Swiss-Prot including isoforms (February 2022). The parameters for the search included: Protein and Precursor Qvalue Cutoff (Experiment) were set to 0.01, with the Protein Qvalue Cutoff (Run) set to 0.05. Quantity MS Level was set to MS2, Protein LFQ Method was set to 'Quant 2.0', the Major Group Quantity was set to the Sum of peptide quantity with the Major Group Top N being disabled. The Minor Group Quantity was to 'Sum of precursor quantity' and the Minor Group Top N was also disabled. Estimated protein and copy number estimates generated were calculated from the MS data using the 'Proteomic Ruler' (Wiśniewski et al. 2014). Finally, differential expression using multiple testing correction was estimated using *limma* (Ritchie et al. 2015) and plotted in Prism (GraphPad).

Cross linked IP mass spectrometry

For mass spectrometry analysis of DSG-crosslinked immunoprecipitation samples, the reduced and alkylated eluates were purified by SDS-PAGE, subjected to in-gel tryptic digestion prior to desalting (C18 column) and 6-plex TMT labelling using TMTsixplex™ Isobaric Label Reagent set (ThermoFisher) according to manufacturer's instructions. The pooled and desalted samples were run on an Orbitrap Fusion Lumos Tribrid spectrometer (ThermoFisher) with SPS-MS3 workflow.

Native IP mass spectrometry

For mass spectrometry analysis of uncrosslinked PROSER1-C immunoprecipitation samples, the reduced and alkylated eluates were purified by SDS-PAGE and subjected to in-gel tryptic digestion as described above. Eluted peptides were analysed on a Orbitrap Exploris 480 Mass Spectrometer (ThermoFisher) using a 120 min gradient elution and data-independent acquisition.

Native IP data analysis

Raw data were processed using Spectronaut® software using the parameters specified above. Differential enrichment of with multiple testing correction was determined using Perseus 1.6.14.0 (Tyanova et al. 2016) and plotted in Prism.

Histone modifications mass spectrometry

Histone modification analysis by mass spectrometry was performed as detailed described in (Joseph-Chowdhury et al. 2022). Cells were harvested and the histones extracted as follows. Cells were incubated in 0.2 M H_2SO_4 for 3 hr at 4°C with rotation, then centrifuged at 3400 xg for 5 min. Trichloroacetic acid was added to the supernatant to a final concentration of 33% (v/v) and incubated overnight at 4°C with rotation. Samples were centrifuged at 3400 xg for 5 min and the pellet washed with acetone and 0.1% (v/v) HCl, followed by acetone alone. The histones were dried, then subjected to propionylation as follows. Samples were resuspended in 15-20% acetonitrile in 100 mM ammonium bicarbonate. Propionic anhydride was added, followed by NH_4OH. The pH was measured and corrected to ~pH 8 if necessary, followed by incubation at room temp for 5 min. The addition of propionic anhydride, NH_4OH and incubation was repeated, and the samples dried. Histones were digested by trypsin overnight and propionylation was repeated on the digested peptides. Finally, the samples were desalted as follows. Oasis HLB resin (Waters) was suspended in acetonitrile at 50 mg/mL and added to a filter plate (Orochem). Vacuum was applied and the flow through discarded. The resin was washed with 0.1% trifluoroacetic acid (TFA), then the samples resuspended in 0.1% TFA and loaded into the plate. Samples were washed with 0.1% TFA, then eluted in 60% acetonitrile/0.1% TFA. The elutes was dried and resuspended in 0.1% TFA, then analysed by LC-MS/MS.

Samples were loaded onto a Dionex RSLC Ultimate 300 (Thermo Scientific), coupled online with an Orbitrap Fusion Lumos (Thermo Scientific). Chromatographic separation was performed with a two-column system, consisting of a C-18 trap cartridge (300 µm ID, 5 mm length) and a picofrit analytical column (75 µm ID, 25 cm length) packed in-house with reversed-phase Repro-Sil Pur C18-AQ 3 µm resin. Peptides were separated using a 30 min gradient from 1-30% buffer B (buffer A: 0.1% formic acid, buffer B: 80% acetonitrile + 0.1% formic acid) at a flow rate of 300 nL/min. The mass spectrometer was set to acquire spectra in a data-independent acquisition (DIA) mode. Briefly, the full

MS scan was set to 300-1 100 m/z in the orbitrap with a resolution of 120 000 (at 200 m/z) and an AGC target of $5x10^5$. MS/MS was performed in the orbitrap with sequential isolation windows of 50 m/z with an AGC target of $2x10^5$ and an HCD collision energy of 30.

Histone peptides raw files were imported into EpiProfile 2.0 software (Yuan et al. 2018). From the extracted ion chromatogram, the area under the curve was obtained and used to estimate the abundance of each peptide. To achieve the relative abundance of post-translational modifications (PTMs), the sum of all different modified forms of a histone peptide was considered as 100% and the area of the particular peptide was divided by the total area for that histone peptide in all of its modified forms. The relative ratio of two isobaric forms was estimated by averaging the ratio for each fragment ion with different mass between the two species. The resulting peptide lists generated by EpiProfile were exported to Microsoft Excel and further processed for a detailed analysis.

Quantification of 5hmdC & 5mdC by LC-MS/MS

Genomic DNA was extracted from frozen cell pellets using the Monarch Genomic DNA Purification kit (New England Biolabs #T3010) and eluted in LC-MS grade water. DNA was digested into nucleosides as described previously (Stewart-Morgan et al. 2023). DNA nucleosides were analysed on an Agilent 1290 UHPLC system with a ZORBAX Eclipse Plus C18 Rapid Resolution HD 2.1x100 mm 1.8-Micron column (Agilent #959758-902) connected to an Agilent 6495B triple quadrupole instrument run in positive ionisation mode. The chromatographic method, mass spectrometer parameters and transitions detected by multiple reaction monitoring were outlined previously (Stewart-Morgan et al. 2023). The quantification was conducted in MassHunter Quantitative Analysis for QQQ (version 10.1) based on a standard curve for each of the DNA nucleosides and the addition of heavy labelled internal standards (Amouroux et al. 2016; Stewart-Morgan et al. 2023). The limit of detection was 0.025 fmol for 5hmdC, 0.005 fmol 5mdC, 0.1 fmol for dC and 0.1 fmol for dG. The lower limit of quantification was 0.025 fmol for 5hmdC, 0.25 fmol 5mdC, 0.1 fmol for dC and 0.5 fmol for dG.

ChIP-seq

Cells were harvested and double crosslinked (DSG+FA) by resuspension in PBS supplemented with 2 mM DSG and incubation at room temp for 30 min, followed by addition of formaldehyde to a final concentration of 1% and a further 10 min incubation. Quenching was performed by addition of glycine to a final concentration of 125 mM. Cells were washed with PBS and lysed by resuspension in SDS buffer: 50 mM Tris-HCl pH 8.1, 100 mM NaCl, 5 mM EDTA, 0.5% SDS supplemented with 1 mM PMSF. The cell contents were collected by centrifugation at 300 xg, 6 min, 20°C and resuspended in IP buffer: 50 mM Tris-HCl pH 8.6, 100 mM NaCl, 5 mM EDTA, 1.6% Triton X-100, 0.3% SDS. Sonication was carried

out using a bioruptor Pico and fragmentation conditions (to obtain 100-500 bp fragments) were optimised for each experiment before continuing. Sonicated samples were cleared by centrifugation at 20 000 xg, 20 min, 4°C and the pellet discarded. Protein concentration was estimated by Bradford assay (BioRad) and the concentrations normalised across samples. SDS-free buffer: 50 mM Tris-HCl pH 8.1, 100 mM NaCl, 5 mM EDTA was used to dilute SDS in samples to a final concentration of 1% and the samples were precleared by 3 hr incubation with protein G sepharose beads.

Immunoprecipitation was carried out overnight on 600 µg protein (TET2, PROSER1) or 60 µg (histone marks). Antibody quantities were as follows: PROSER1: PROSER1-N antibody (Sheep polyclonal (this study), 1.2 µg antibody to 600 µg chromatin), TET2: TET2-N antibody (Rasmussen et al. 2019) (Rabbit polyclonal, 1 µg antibody for 600 µg chromatin), H3K4me1: CST 5326 (Rabbit monoclonal, 10 µL to 60 µg chromatin), H3K27ac: ThermoFisher MA5-23516 (Mouse monoclonal, 4 µg antibody to 60 µg chromatin)). Protein G sepharose beads were used to bind the antibody and were washed three times with low salt wash buffer: 20 mM Tris-HCl pH 8.0, 150 mM NaCl, 2 mM EDTA, 0.1% SDS, 1% Triton X-100, twice with high salt wash buffer: 20 mM Tris-HCl pH 8.0, 500 mM NaCl, 2 mM EDTA, 0.1% SDS, 1% Triton X-100, and once with IP buffer with a final concentration of 0.1% SDS. Beads were resuspended in decrosslinking buffer: 1% SDS and 100 mM $NaHCO_3$ and incubated at 65°C, shaking at 1200 RPM overnight. DNA was purified using the Monarch PCR & DNA cleanup kit. Libraries were prepared from 1-3 ng purified DNA input using Truseq adapters from Illumina and sequenced with on a NovaSeq 6000 system with 150 bp paired-end sequencing.

ChIP-seq computational analysis

Paired-end Illumina reads obtained by sequencing (PROSER1, TET2, H3K27ac, H3K37me3 and H3K4me1) or from Gene Expression Omnibus (GEO) (TET1 (GSE24843), CTCF (GSE123670), OGT (GSE39154), PSPC1 (GSE182442), P300 (GSE97701), SUZ12 (GSE103685)) were trimmed of adapter sequences and low-quality nucleotides using Trim Galore! (CTCF and OGT) or Trimmomatic (all other datasets), then mapped to the mouse genome (mm10) using Bowtie2 (Langmead and Salzberg 2012) with (--very-sensitive) preset settings. MarkDuplicates from Picard tools was used to de-duplicate mapped reads. Mapped reads were filtered to remove those from non-chromosomal DNA, mtDNA, and the Y chromosome. Peaks were called using MACS2 with paired-end settings (--format BAMPE --mfold 5 50 --bw 300) using a 0.05 p-adj threshold for OGT, CTCF, TET2 and PROSER1 peaks, a 0.01 threshold for TET1 and a 0.001 threshold for PSPC1, H3K4me1, H3K27me3 and H3K27ac. In addition, H3K4me1 and H3K27me3 peaks were called as broad, with a broad region cutoff of 0.1. Differential binding across replicates was defined using DiffBind (Stark R 2011). For visualization, BigWig coverage files normalised to sequencing depth (Reads per bin/(number of mapped reads (millions))*100) were

generated from merged BAM files using bamCoverage with the following configuration (--binSize 10 --normalizeUsing RPKM --extendReads --ignoreDuplicates –blackListFileName 'mm10-blacklist.v2.bed.gz'). Heatmaps were generated using deepTools (Ramírez et al. 2014) and Volcano plots and frequency histograms were generated and plotted with Prism.

To investigate PROSER1 genomic localisation, regionsets were obtained for CpG islands (CGI), transcription start sites (TSS), promoters, active enhancers and gene bodies. Datasets defining CGI, TSS, gene bodies were obtained from UCSC table browser (GRCm38/mm10) and heterochromatic regions were obtained from ChromHMM annotation (https://github.com/guifengwei/ChromHMM_mESC_mm10). Promoters were defined as -1500 bp to +500 bp from a TSS. Active enhancers were defined by the presence of both H3K4me1 and H3K27ac and excluding regions overlapping promoters or CGIs. Non-CGI promoters were defined as promoters which had no overlap with CGIs. Matched control peaksets were generated using EaSeq (Lerdrup et al. 2016) 'Matched Controls' tool and matched to DNAse hypersensitive sites in mESCs. Overlaps between datasets were assessed using intersect intervals from Bedtools (Quinlan and Hall 2010).

To calculate fold change in histone modifications or protein binding within PROSER1 or TET2 peaks, the peakset was concatenated with DNaseI hypersensitivity sites in mESCs (obtained from ENCODE portal ENCFF048DWN) to provide a genome background regionset for estimation of dispersion and subsequent normalisation. Using FeatureCounts (Liao et al. 2014) with multi-mapping and multi-overlapping features allowed, reads from histone modification, TET2 or PROSER1 ChIP-seq that overlapped with any region in the peakset+DNaseHS regionset were summarized. Finally, Log_2 fold changes of normalised read counts within the regionset was obtained using DESeq2 (Love et al. 2014) and plotted with Prism. Computational analysis was performed through the free public European Galaxy server usegalaxy.eu (Community 2024).

Enzymatic Methyl-seq (EM-seq)

Two biological replicate samples each of WT, PROSER1 KO1 and PROSER1 KO+Rescue mESCs were harvested and gDNA extracted using the Monarch Genomic DNA Purification Kit (New England Biolabs) to preserve DNA integrity prior to sonication. Sonication was carried out using a bioruptor Pico (Diagenode) and fragmentation conditions (to obtain 300-450 bp fragments) were optimised for each experiment before continuing. EM-seq was performed using the NEBNext Enzymatic Methyl-seq Kit (New England Biolabs) according to the protocol for use with Large Insert Libraries using 100 ng input DNA.

Enzymatic Methyl-seq (EM-seq) computational analysis

Raw EM-seq reads from the two biological replicate WT, PROSER1 KO, and PROSER1 KO+Rescue mESC samples were trimmed using TrimGalore and mapped against the mouse genome (mm10) using Bismark (https://github.com/FelixKrueger/Bismark) with standard setting allowing for 1000 bp insert size (--X 1000). Bismark deduplicate was used to remove PCR duplicates. Bismark methylation extractor was used with the following parameter setting (--paired-end --no-overlap --ignore_r2 3 --ignore_3prime_r2 1) to remove methylation call bias due to adaptor ligation and avoid scoring methylation calls twice in case of overlapping paired-end reads. Methylation calls in a CpG context were merged (combining CpG methylation calls from both strands of a single CG dinucleotide) to produce a comprehensive 'Methreport_CpGcomb_mm10' file containing summarised methylation calls for all CG sites in the mouse genome. All analysis was performed through the free public European Galaxy server usegalaxy.eu (Community 2024).

Individual methylation calls from each biological replicate were imported into SeqMonk NGS visualisation and analysis tool (https://github.com/s-andrews/SeqMonk) using the QuasR import filter and input files with the following columns <chr><start><end><total reads><methylated reads>. Following a correlation analysis to determine reproducibility between biological replicates, the datasets were pooled to attain a collective coverage of ~17.5 million CpG sites, each supported by a minimum of 10 distinct EM-seq reads across all three genotypic groups – a condition required for all downstream analysis. To define 10 kb genomic tiles the running window probe generator was used with a probe and step size of 10,000 bp. Quantitation trend plots of different genomic elements were generated using a 50 bp window and 50 bp step size as well as 1kb flanking sequence. Significantly differentially methylated CpG sites were defined using the Chi-squared filter, only retaining sites with p-adj<0.05 after multiple testing correction and a minimum 25% difference in methylation value. Data were exported from SeqMonk and visualized using Prism.

RNA-seq

Two biological replicate samples each of WT, PROSER1 KO1 and PROSER1 KO+Rescue mESCs were subjected to monolayer neuronal differentiation (Pantier et al. 2021). Briefly, they were trypsinised and washed once in PBS, then replated on gelatinised 6-well plates at a density of 100 000 cells/well in N2B27 medium: 1:1 mix of Advanced DMEM-F12 and Neurobasal, supplemented with 1x L-Glutamine, 1x NEAA, 0.5x N-2 supplement, 0.5x B-27 supplement, and 0.1 mM β-ME (all supplied by Gibco). RNA was extracted at day 0 (when cells were first plated in N2B27 medium) and at day 2 using the QIAGEN RNeasy kit. RNA integrity was checked by gel electrophoresis on an Agilent Tapestation system. Libraries were prepared using the NEBNext Ultra II Directional RNA Library Prep kit (NEB).

RNA-seq computational analysis

To visualise changes in protein coding genes, raw RNA-seq reads from the biological replicate WT, PROSER1 KO, and PROSER1 KO+Rescue mESC samples were trimmed using Trimmomatic and mapped to the mouse genome (mm10) using RNA-STAR (Dobin et al. 2013) with paired-end settings. Gene annotation 'mm10.ncbiRefSeq.gtf.gz' was obtained from RefSeq (O'Leary et al. 2016) and gene expression was summarized from the mapped reads using FeatureCounts, with option (--countReadPairs) to enable counting of fragments from paired-end data. Mapped reads were filtered to remove those from the Y chromosome. Differential expression was determined using DESeq2 with default parameters. Visualisation of RNA-seq counts within IGV tracks were achieved by merging BAM files from replicate samples and generating bigwig files with BamCoverage from deepTools using the following parameters as well as removal of blacklisted regions (--binSize 10 --normalizeUsing RPKM --extendReads --blackListFileName 'mm10-blacklist.v2.bed.gz').

TET2 binding region-gene associations were determined using 'Basal plus extension' in GREAT (McLean et al. 2010). In short, each gene was assigned a 5kb upstream and 1kb downstream basal regulatory domain, extending to nearest neighbouring basal domain but no more than 1Mb in one direction. TET2 binding regions were then linked to genes overlapping this domain resulting in 866 genes (see Supplemental Tab. S7) associated with loss of TET2 binding (p-adj<0.05, $Log_2FC<-1$) and 3341 genes associated with gain of TET2 binding (p-adj<0.05, $Log_2FC>1$) that were used for GREAT gene ontology enrichment analysis. The z-scores of the resulting genes during neuronal differentiation were furthermore visualised and clustered in heatmaps using MeV (Multiple Experiment Viewer) 4.9.0 (SourceForge).

To visualise changes in TEs, raw RNA-seq reads from the biological replicate WT, PROSER1 KO, and PROSER1 KO+Rescue mESC samples were trimmed as above and mapped to the mouse genome (mm10) using RNA-STAR with parameter settings to retain multimapping reads (--outFilterMultimapNmax 100, and --winAnchorMultimapNmax 100). Mapped reads were filtered to remove those from the Y chromosome. Mapped reads were subsequently employed as input for the TEtranscripts pipeline, utilizing the '-multi' parameter as well as TE GTF file 'mm10_rmsk_TE.gtf.gz', available from the software authors. Prior to differential expression analysis using DESeq2, lowly expressed transposable elements (TEs) with a read count below 25 were filtered out (--minread 25). MA-plot showing differential expression of TE families was generated with Prism. To examine differential expression of individual TEs solely based on uniquely mapped reads, trimmed read data were aligned to the mm10 mouse genome reference using STAR with the parameters modified to retain only uniquely mapped reads (--outFilterMultimapNmax 1, and --winAnchorMultimapNmax 50). Mapped reads were subsequently counted using FeatureCounts and the TE GTF file

‘mm10_rmsk_TE.gtf.gz’ as well as option (--countReadPairs) to enable counting of fragments from paired-end data. Differential expression was then determined using DESeq2 with default parameters. Expression values of all members of selected TE families were extracted and box plots comparing PROSER1 KO vs WT and KO+Rescue vs WT were generated with Prism. All analysis was performed through the free public European Galaxy server usegalaxy.eu (Community 2024).

References

Amouroux R, Nashun B, Shirane K, Nakagawa S, Hill PWS, D’Souza Z, Nakayama M, Matsuda M, Turp A, Ndjetehe E, et al. 2016. De novo DNA methylation drives 5hmC accumulation in mouse zygotes. *Nat Cell Biol* **18**: 225–233.

Community TG. 2024. The Galaxy platform for accessible , reproducible , and collabor ativ e data analyses : 2024 update.

Dobin A, Davis CA, Schlesinger F, Drenkow J, Zaleski C, Jha S, Batut P, Chaisson M, Gingeras TR. 2013. STAR: Ultrafast universal RNA-seq aligner. *Bioinformatics* **29**: 15–21.

Højfeldt JW, Laugesen A, Willumsen BM, Damhofer H, Hedehus L, Tvardovskiy A, Mohammad F, Jensen ON, Helin K. 2018. Accurate H3K27 methylation can be established de novo by SUZ12-directed PRC2. *Nat Struct Mol Biol* **25**: 225–232.

Hon GC, Song C-X, Du T, Jin F, Selvaraj S, Lee AY, Yen C-A, Ye Z, Mao S-Q, Wang B-A, et al. 2014. 5mC oxidation by Tet2 modulates enhancer activity and timing of transcriptome reprogramming during differentiation. *Mol Cell* **56**: 286–297.

Huang X, Bashkenova N, Hong Y, Lyu C, Guallar D, Hu Z, Malik V, Li D, Wang H, Shen X, et al. 2022. A TET1-PSPC1-Neat1 molecular axis modulates PRC2 functions in controlling stem cell bivalency. *Cell Rep* **39**: 110928.

Joseph-Chowdhury JSN, Stransky S, Graff S, Cutler R, Young D, Kim JS, Madrid-Aliste C, Aguilan JT, Nieves E, Sun Y, et al. 2022. Global Level Quantification of Histone Post-Translational Modifications in a 3D Cell Culture Model of Hepatic Tissue. *J Vis Exp* **2022**.

Langmead B, Salzberg SL. 2012. Fast gapped-read alignment with Bowtie 2. *Nat Methods* **9**: 357–359.

Lerdrup M, Johansen JV, Agrawal-Singh S, Hansen K. 2016. An interactive environment for agile analysis and visualization of ChIP-sequencing data. *Nat Struct Mol Biol* **23**: 349–357.

Liao Y, Smyth GK, Shi W. 2014. FeatureCounts: An efficient general purpose program for assigning sequence reads to genomic features. *Bioinformatics* **30**: 923–930.

Love MI, Huber W, Anders S. 2014. Moderated estimation of fold change and dispersion for RNA-seq data with DESeq2. *Genome Biol* **15**: 1–21.

McLean CY, Bristor D, Hiller M, Clarke SL, Schaar BT, Lowe CB, Wenger AM, Bejerano G. 2010. GREAT improves functional interpretation of cis-regulatory regions. *Nat Biotechnol* **28**: 495–501.

O’Leary NA, Wright MW, Brister JR, Ciufo S, Haddad D, McVeigh R, Rajput B, Robbertse B, Smith-White B, Ako-Adjei D, et al. 2016. Reference sequence (RefSeq) database at NCBI: Current status, taxonomic expansion, and functional annotation. *Nucleic Acids Res* **44**: D733–D745.

Pantier R, Chhatbar K, Quante T, Skourti-Stathaki K, Cholewa-Waclaw J, Alston G, Alexander-Howden B, Lee HY, Cook AG, Spruijt CG, et al. 2021. SALL4 controls cell fate in response to DNA base composition. *Mol Cell* **81**: 845-858.e8.

Quinlan AR, Hall IM. 2010. BEDTools: A flexible suite of utilities for comparing genomic features. *Bioinformatics* **26**: 841–842.

Ramírez F, Dündar F, Diehl S, Grüning BA, Manke T. 2014. DeepTools: A flexible platform for exploring deep-sequencing data. *Nucleic Acids Res* **42**: 187–191.

Rasmussen KD, Berest I, Keβler S, Nishimura K, Simón-Carrasco L, Vassiliou GS, Pedersen MT, Christensen J, Zaugg JB, Helin

K. 2019. TET2 binding to enhancers facilitates transcription factor recruitment in hematopoietic cells. *Genome Res* **29**: 564–575.

Ritchie ME, Phipson B, Wu D, Hu Y, Law CW, Shi W, Smyth GK. 2015. Limma powers differential expression analyses for RNA-sequencing and microarray studies. *Nucleic Acids Res* **43**: e47.

Song Y, Liang Z, Zhang J, Hu G, Wang J, Li Y, Guo R, Dong X, Babarinde IA, Ping W, et al. 2022. CTCF functions as an insulator for somatic genes and a chromatin remodeler for pluripotency genes during reprogramming. *Cell Rep* **39**: 110626.

Stark R, Brown G (2011). *DiffBind: differential binding analysis of ChIP-Seq peak data*. http://bioconductor.org/packages/release/bioc/vignettes/DiffBind/inst/doc/DiffBind.pdf.

Stewart-Morgan KR, Requena CE, Flury V, Du Q, Heckhausen Z, Hajkova P, Groth A. 2023. Quantifying propagation of DNA methylation and hydroxymethylation with iDEMS. *Nat Cell Biol* **25**: 183–193.

Tyanova S, Temu T, Sinitcyn P, Carlson A, Hein MY, Geiger T, Mann M, Cox J. 2016. The Perseus computational platform for comprehensive analysis of (prote)omics data. *Nat Methods* **13**: 731–740.

Vella P, Scelfo A, Jammula S, Chiacchiera F, Williams K, Cuomo A, Roberto A, Christensen J, Bonaldi T, Helin K, et al. 2013. Tet proteins connect the O-linked N-acetylglucosamine transferase Ogt to chromatin in embryonic stem cells. *Mol Cell* **49**: 645–656.

Wang SP, Tang Z, Chen CW, Shimada M, Koche RP, Wang LH, Nakadai T, Chramiec A, Krivtsov A V., Armstrong SA, et al. 2017. A UTX-MLL4-p300 Transcriptional Regulatory Network Coordinately Shapes Active Enhancer Landscapes for Eliciting Transcription. *Mol Cell* **67**: 308-321.e6..

Williams K, Christensen J, Pedersen MT, Johansen J V, Cloos PAC, Rappsilber J, Helin K. 2011. TET1 and hydroxymethylcytosine in transcription and DNA methylation fidelity. *Nature* **473**: 343–348.

Wiśniewski JR, Hein MY, Cox J, Mann M. 2014. A “proteomic ruler” for protein copy number and concentration estimation without spike-in standards. *Mol Cell Proteomics* **13**: 3497–3506.

Yuan ZF, Sidoli S, Marchione DM, Simithy J, Janssen KA, Szurgot MR, Garcia BA. 2018. EpiProfile 2.0: A Computational Platform for Processing Epi-Proteomics Mass Spectrometry Data. *J Proteome Res* **17**: 2533–2541.
